# Comparative Proteomic Analysis of tPVAT during Ang II Infusion

**DOI:** 10.3390/biomedicines9121820

**Published:** 2021-12-02

**Authors:** Xiuying Liang, Haijing Guan, Jingwen Sun, Yan Qi, Wenjuan Yao

**Affiliations:** Department of Pharmacology, School of Pharmacy, Nantong University, 19 QiXiu Road, Nantong 226001, China; 1617042068@stmail.ntu.edu.cn (X.L.); 1627012011@stmail.ntu.edu.cn (H.G.); 1727011021@stmail.ntu.edu.cn (J.S.); 1923310019@stmail.ntu.edu.cn (Y.Q.)

**Keywords:** perivascular adipose tissue, renin-angiotensin system, angiotensin II, proteomic analysis, tandem mass tags

## Abstract

Perivascular adipose tissue (PVAT) homeostasis plays an important role in maintaining vascular function, and PVAT dysfunction may induce several pathophysiological situations. In this study, we investigated the effect and mechanism of the local angiotensin II (Ang II) on PVAT. High-throughput comparative proteomic analysis, based on TMT labeling combined with LC-MS/MS, were performed on an in vivo Ang II infusion mice model to obtain a comprehensive view of the protein ensembles associated with thoracic PVAT (tPVAT) dysfunction induced by Ang II. In total, 5037 proteins were confidently identified, of which 4984 proteins were quantified. Compared with the saline group, 145 proteins were upregulated and 146 proteins were downregulated during Ang II-induced tPVAT pathogenesis. Bioinformatics analyses revealed that the most enriched GO terms were annotated as gene silencing, monosaccharide binding, and extracellular matrix. In addition, some novel proteins, potentially associated with Ang II infusion, were identified, such as acyl-CoA carboxylase α, very long-chain acyl-CoA synthetase (ACSVL), uncoupling protein 1 (UCP1), perilipin, RAS protein-specific guanine nucleotide-releasing factor 2 (RasGRF2), and hypoxia inducible factor 1α (HIF-1α). Ang II could directly participate in the regulation of lipid metabolism, transportation, and adipocyte differentiation by affecting UCP1 and perilipin. Importantly, the key KEGG pathways were involved in fatty acid biosynthesis, FABP3-PPARα/γ, RasGRF2-ERK-HIF-1α, RasGRF2-PKC-HIF-1α, and STAT3-HIF-1α axis. The present study provided the most comprehensive proteome profile of mice tPVAT and some novel insights into Ang II-mediated tPVAT dysfunction and will be helpful for understanding the possible relationship between local RAS activation and PVAT dysfunction.

## 1. Introduction

The renin–angiotensin–aldosterone system (RAAS) is involved in systemic blood pressure regulation and renal electrolyte homeostasis. It is well accepted that Ang II mediates vascular smooth muscle cell (VSMC) proliferation, migration, and vascular remodeling [1,2]. We also previously reported that Ang II regulates VSMC proliferation and vascular remodeling by RhoGDI signaling [3]. However, there is no detailed report on the effect of Ang II on PVAT (perivascular adipose tissue) structure and function.

PVAT is an adipose tissue with endocrine and paracrine functions surrounding the blood vessels, such as the small mesenteric and femoral arteries or the large abdominal aorta (AA), and plays wide-ranging physiological roles on vascular environment stability and influences vascular pathogenesis [4,5]. Under physiological conditions, PVAT has strong anti-atherosclerosis properties, induces non-shivering thermogenesis (NST), and metabolizes fatty acids by secreting various bioactive factors [6]. Under pathological conditions (such as obesity), PVAT loses its thermogenic ability and secretes pro-inflammatory adipocytokines that induce endothelial dysfunction and inflammatory cell infiltration and promotes the development of atherosclerosis [6]. The PVAT in the thoracic aorta is more similar to brown adipose tissue (BAT), while the PVAT in the abdominal aorta is similar to white adipose tissue (WAT). WAT primarily stores energy in the form of triglycerides, while excessive lipid accumulation in WAT leads to adipocyte hypertrophy and dysfunction. This leads to increased secretion of harmful fat factors and inflammatory cytokines into circulation, thus impairing the function of the vascular endothelium [7,8]. Unlike WAT, BAT can absorb lipids by uncoupling oxidation in the mitochondrial electron transport chain to generate heat, and this is characterized by the expression of the heat-producing gene uncoupling protein 1 (UCP1) [9]. In addition, WAT can be browned under the stimulation of low temperature and catecholamine [10,11]. The browning of WAT is characterized by a high expression of the brown adipocyte marker UCP1 [12], which is now called the third adipose tissue-beige adipose tissue (BEt). In general, changes in the phenotype of PVAT correlate with disease progression.

Many studies have shown that there is local RAAS activity in PVAT [13]. It has been reported that Ang II inhibits adipogenic differentiation of human preadipocytes in vitro via the AT1 receptor [14]. The AngII-AT2 or Ang (1–7)-Mas activation stimulates adipogenesis and antagonizes the antiadipogenic effect of AngII-AT1 activation [15,16]. However, the effect of Ang II on PVAT remains unclear. In this study, we used TMT, combined with LC-MS/MS and bioinformatics analysis, to analyze the phenotypic changes of PVAT during Ang II infusion.

## 2. Materials and Methods

### 2.1. Materials

Ang II was obtained from MedChemExpress (#HY-B0202; Princeton, NJ, USA). The primary antibodies against KDM1A (BM4356), ALCAM (A01788-1), MBNL1 (A02309-1), and TSN (A02777) were purchased from Boster Biological Technology (Wuhan, Hubei, China). Anti-TMEM41B (29270-1-AP), -PRTN3 (25278-1-AP), -GIGYF2 (24790-1-AP), -NHP2 (15128-1-AP), -RDH10 (14644-1-AP), and -TIMD4 (12008-1-AP) antibodies were purchased from Proteintech (Chicago, IL, USA). Anti-CtBP1 (ab129181), -PKN1 (ab231038), and -CCN1 (ab228592) antibodies were purchased from Abcam (Cambridge, MA, USA). Peroxidase-conjugated AffiniPure goat anti-rabbit IgG (H + L) was from Proteintech (SA00001–2; Chicago, IL, USA). An SABC immunohistochemistry staining kit was sourced from BosterBio (SA1028; Wuhan, Hubei, China). All other chemicals used in this study were analytical grade and were made in China.

### 2.2. Animals and Experimental Protocol

Animal procedures were performed in accordance with the Ethics Committee and the Animal Care and Use Committee of Nantong University (Ethic Committee approval number: 1213201.1) and conformed to the NIH Guide for the Care and Use of Laboratory Animals. Male C57BL/6 mice, aged 45 days weighing 20 ± 1 g (20 mice per group), were purchased from Beijing Vital River Laboratory Animal Technology Co., Ltd. (Beijing, China). Mice were anesthetized by intraperitoneal injection of 3.6% chloral hydrate (11 mL/kg). For Ang II infusion, mice were implanted with an Alzet Model 1002 osmotic minipump (Alzet Corp, Cupertino, CA, USA) for subcutaneous infusion of Ang II at a rate of 1000 ng/kg/min [3]. Control mice were implanted with pumps for infusion of normal saline. Pumps were placed into the subcutaneous space of anesthetized mice through a small incision in the back of the neck. The incision was closed, and the mice were allowed to recover without medication. After 14 days, mice were then euthanized. The aortic tree was quickly separated and washed with pre-chilled saline for subsequent analysis.

### 2.3. Adipose Tissue Transplantation

The PVAT around the thoracic aorta was carefully removed with microforceps under a surgical microscope. The removed thoracic PVAT (tPVAT) was then weighed and placed in Dulbecco’s Modifed Eagle Medium (DMEM, Gibco, Termo Fisher Scientifc, Inc., Waltham, MA, USA) containing 1% antibiotics (R&D Systems, Inc., Minneapolis, MN, USA). For proteomics analysis, the normal and Ang II infusion group had no less than 600 mg of the removed tPVAT in each group. The remaining tPVAT and blood vessels were used for the following experiments or stored in a liquid nitrogen tank.

### 2.4. H&E Staining

The removed tPVAT around the thoracic aorta was fixed in 4% paraformaldehyde and then embedded in paraffin for staining with hematoxylin and eosin. For quantitative analysis of adipocyte diameter, tissue sections were captured with Olympus digital camera (Tokyo, Japan) and quantified via Image-Pro Plus 6.0 software (Media Cybernetics, Rockville, MD, USA).

### 2.5. Immunohistochemical Analysis

Immunohistochemical staining against KDM1A, ALCAM, MBNL1, TSN, TMEM41B, PRTN3, GIGYF2, NHP2, RDH10, TIMD4, CtBP1, PKN1, and CCN1 were performed using a strept avidin-biotin complex (SABC) immunohistochemistry staining kit and following the manufacturer’s instructions. Paraffin-embedded tissue sections of the transplanted tPVAT (5 μm thick) were deparafnized and blocked with 0.5% horse serum. The sections were then incubated with the primary antibodies overnight at 4 °C, followed by incubation with biotinylated anti-rabbit IgG as the secondary antibody at room temperature for 2 h and SABC for 1 h. The samples were then visualized using a diaminobenzidine (DAB) staining kit, followed by counterstaining with hematoxylin, in order to stain the target proteins brown. All images were captured using an Olympus digital camera (Olympus, Tokyo, Japan) and analyzed using the Image-Pro Plus 6.0 software program (Media Cybernetics, Rockville, MD, USA).

### 2.6. Quantitative Proteomic Profiling by Tandem Mass Tag (TMT) Technology

**Figure 1:**
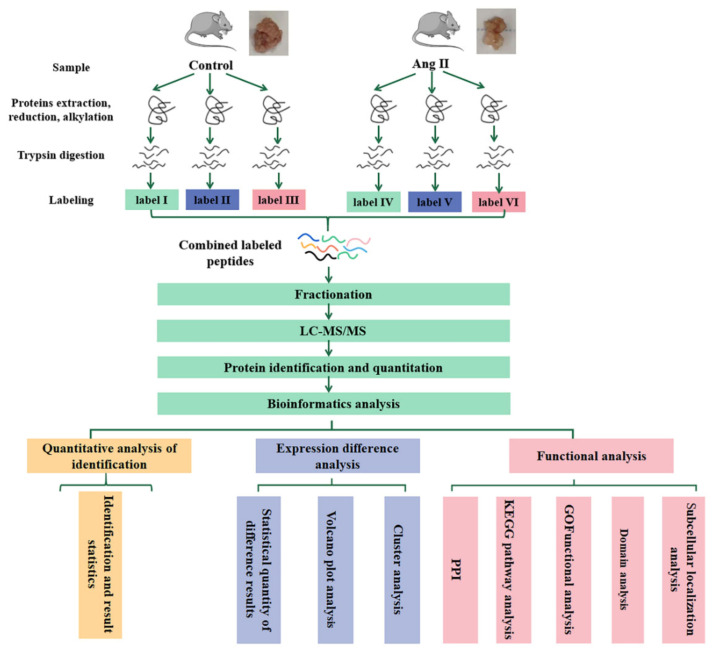
Workflow for the whole study. tPVAT samples in each group (no less than 600 mg per group) were subjected to three biological replicates (each replicate is a mixture of tPVAT from 5–7 mice). LC-MS/MS, liquid chromatography-tandem mass spectrometry; GO, gene ontology; KEGG, Kyoto Encyclopedia of Genes and Genomes; PPI, Protein Protein Interaction.

The flowchart of proteomics and bioinformatics analysis is shown in Figure 1.

The removed tPVAT around the thoracic aorta was ground to powder in liquid nitrogen. Proteins were extracted in lysis buffer (4% SDS, 100 mM Tris-HCL, 1 mM DTT, pH 7.6) for 30 min on ice. Then, cells were further broken using an ultrasonic cell disruptor, followed by centrifugation at 14,000 rpm for 1.5 h at 19 °C using a TL-100 ultracentrifuge (Beckman, Palo Alto, Brea, CA, USA). Finally, the middle layer of aqueous liquid was retained. The amount of protein was quantified with the BCA Protein Assay Kit (Bio-Rad, Hercules, CA, USA). Protein digestion by trypsin was performed according to filter-aided sample preparation (FASP) procedure described previously [17]. The digest peptides of each sample were desalted on C18 Cartridges (Empore™ SPE Cartridges C18, Oxford, PA, USA (standard density), bed I.D. 7 mm, volume 3 mL, Sigma), concentrated by vacuum centrifugation and reconstituted in 40 µL of 0.1% (*v*/*v*) formic acid. The peptide content was estimated by UV light spectral density at 280 nm using an extinctions coefficient of 1.1 of 0.1% (g/L) solution that was calculated on the basis of the frequency of tryptophan and tyrosine in vertebrate proteins.

100 μg peptide mixture of each sample was labeled using TMT reagent according to the manufacturer’s instructions (Thermo Scientific, Waltham, MA, USA). Labeled peptides were fractionated by SCX chromatography using the AKTA Purifier system (GE Healthcare, Chicago, IL, USA). The dried peptide mixture was reconstituted and acidified with buffer A (10 mM KH_2_PO_4_ in 25% of ACN, pH 3.0) and loaded onto a PolySULFOETHYL 4.6 × 100 mm column (5 µm, 200 Å, PolyLC Inc., Columbia, MD, USA). The peptides were eluted at a flow rate of 1 mL/min with a gradient of 0% buffer B (500 mM KCl, 10 mM KH_2_PO_4_ in 25% of ACN, pH 3.0) for 25 min, 0–10% buffer B during 25–32 min, 10–20% buffer B during 32–42 min, 20–45% buffer B during 42–47 min, 45–100% buffer B during 47–52 min, 100% buffer B during 52–60 min, and buffer B was reset to 0% after 60 min. The elution was monitored by absorbance at 214 nm, and fractions were collected every 1 min. The collected fractions were desalted on C18 Cartridges and concentrated by vacuum centrifugation.

Liquid chromatography tandem mass spectrometry (LC-MS/MS) analysis was performed on a Q Exactive mass spectrometer (Thermo Scientific, Waltham, MA, USA) that was coupled to Easy nLC (Proxeon Biosystems, now Thermo Fisher Scientific) for 60/90 min. The peptides were loaded onto a reverse phase trap column (Thermo Scientific Acclaim PepMap100, 100 μm × 2 cm, nanoViper C18), connected to the C18-reversed phase analytical column (Thermo Scientific Easy Column, 10 cm long, 75 μm inner diameter, 3 μm resin) in buffer A (0.1% Formic acid) and separated with a linear gradient of buffer B (84% acetonitrile and 0.1% Formic acid) at a flow rate of 300 nL/min, controlled by IntelliFlow technology. The mass spectrometer was operated in positive ion mode. MS data was acquired using a data-dependent top-10 method, dynamically choosing the most abundant precursor ions from the survey scan (300–1800 *m*/*z*) for HCD fragmentation. Automatic gain control (AGC) target was set to 3e6, and maximum inject time to 10 ms. Dynamic exclusion duration was 40.0 s. Survey scans were acquired at a resolution of 70,000 at *m*/*z* 200 and resolution for HCD spectra was set to 17,500 at *m*/*z* 200, and isolation width was 2 *m*/*z*. Normalized collision energy was 30 eV and the underfill ratio, which specifies the minimum percentage of the target value likely to be reached at maximum fill time, was defined as 0.1%. The instrument was run with peptide recognition mode enabled.

### 2.7. Identification and Quantitation of Proteins

The MS raw data for each sample were searched using the MASCOT engine (Matrix Science, London, UK; version 2.2), embedded into Proteome Discoverer 1.4 software for identification and quantitation analysis. Related parameters and instructions were shown in Supplementary Materials Table S1. The mass spectrometry proteomics data have been deposited to the ProteomeXchange Consortium via the PRIDE partner repository with the dataset identifier PXD029980.

### 2.8. Bioinformatic Analysis

Upregulated genes and downregulated genes were used to conduct a cluster analysis, a subcellular localization analysis, a Gene Ontology (GO, Valencia, Spain) analysis, a Kyoto Encyclopedia of Genes and Genomes (KEGG, Kyoto, Japan) pathway and enrichment analysis, and a protein-protein interaction (PPI, Hinxton, Cambridgeshire, UK) analysis.

#### 2.8.1. Cluster Analysis

Cluster 3.0 (http://bonsai.hgc.jp/~mdehoon/software/cluster/software.htm, accessed on 17 February 2020) and Java Treeview software 3.0 (http://jtreeview.sourceforge.net, accessed on 17 February 2020) were used to perform hierarchical clustering analysis. Euclidean distance algorithm for similarity measure and average linkage clustering algorithm (clustering uses the centroids of the observations) for clustering were selected when performing hierarchical clustering. A heat map was often presented as a visual aid, in addition to the dendrogram.

#### 2.8.2. Subcellular Localization

CELLO (http://cello.life.nctu.edu.tw/, accessed on 17 February 2020), which is a multi-class SVM classification system, was used to predict protein subcellular localization.

#### 2.8.3. GO Analysis

The protein sequences of the selected differentially expressed proteins (DEPs) were locally searched using the NCBI BLAST+ client software (ncbi-blast-2.2.28 + -win32.exe) and InterProScan to find homologue sequences, then GO terms were mapped and sequences were annotated using the software program Blast2GO (version 2.5.0). The GO annotation results were plotted by R scripts.

#### 2.8.4. KEGG Pathway

Following annotation steps, the studied proteins were blasted against the online KEGG database (http://geneontology.org/, accessed on 17 February 2020) to retrieve their KEGG orthology identifications and were subsequently mapped to pathways in KEGG. Enrichment analyses were applied based on the Fisher’ exact test, considering the whole quantified proteins as background dataset. Benjamini–Hochberg correction for multiple testing was further applied to adjust derived *p*-values. Only functional categories and pathways with *p*-values under a threshold of 0.05 were considered significant.

#### 2.8.5. PPI Analysis

The PPI information of the studied proteins was retrieved from IntAct molecular interaction database (http://www.ebi.ac.uk/intact/, accessed on 17 February 2020) by their gene symbols or STRING software (http://string-db.org/, accessed on 17 February 2020, version 11.5). The results were downloaded in the XGMML format and imported into Cytoscape software (http://www.cytoscape.org/, accessed on 17 February 2020, version 3.2.1) to visualize and further analyze functional protein-protein interaction networks. Furthermore, the degree of each protein was calculated to evaluate the importance of the protein in the PPI network.

### 2.9. Statistical Analysis

All of the results are expressed as the mean ±SD. One-way ANOVA, followed by Tukey’s post-hoc test, as implemented in SPSS 22.0, was used for statistical analysis. Differences with a value of *p* < 0.05 were considered to be statistically significant.

## 3. Results

### 3.1. Pathological Conversion of tPVAT after Ang II Infusion

**Figure 2:**
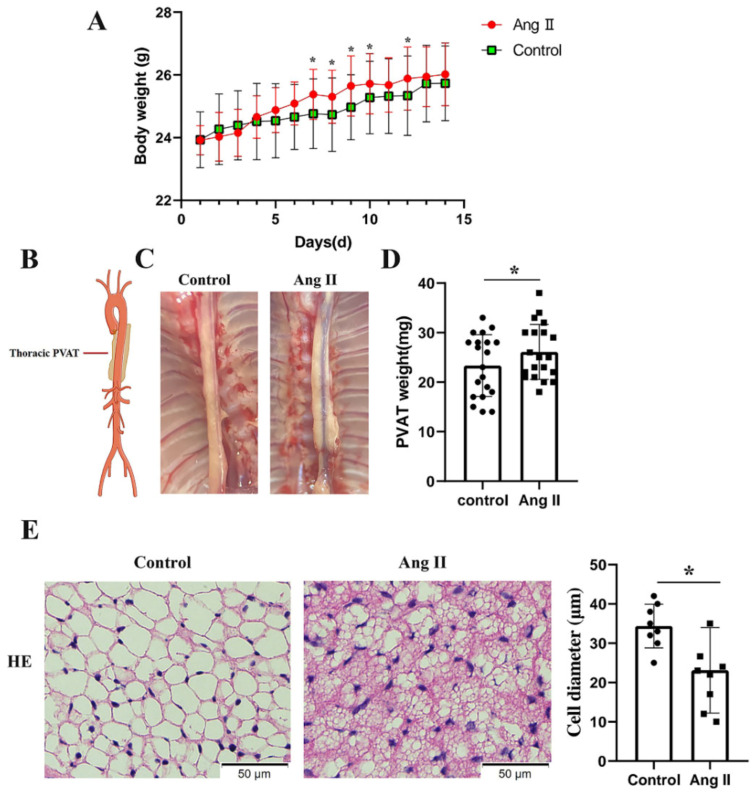
Phenotypic differences between saline and Ang II infused mice. Mice infused with saline were used as control. (**A**) Body weights of the mice perfused with saline and Ang II, during 2 weeks of perfusion (*n* = 20, biological replicates); * *p* < 0.05, vs. the control group (saline infusion). (**B**) Simplified schematic showing thoracic PVAT (tPVAT) in mice; PVAT, perivascular adipose tissue. (**C**) Representative picture illustrating isolation of tPVAT. (**D**) tPVAT weights from saline and Ang II infused mice (*n* = 20, biological replicates); * *p* < 0.05, vs. the control group. (**E**) Hematoxylin and eosin staining of tPVAT and the quantification of cell diameter between saline and Ang II infusion group (*n* = 8, biological replicates); * *p* < 0.05, vs. the control group.

To determine the effects of Ang II on the tPVAT phenotype, male C57BL/6J mice aged 42–48 days were infused with Ang II or normal saline for two weeks. Mice that were infused with Ang II were significantly heavier than the controls by 7 and 12 days (Figure 2A). The tPVAT was successfully separated (Figure 2B,C). Figure 2C shows that Ang II infusion made the tPVAT morphology significantly irregular and caused hypertrophy, when compared with the saline group. The tPVAT weight of the Ang II infusion group was significantly higher than that of the control group (Figure 2D). Next, we histologically analyzed tPVAT after Ang II infusion. Mice infused with Ang II had a marked phenotypic conversion of tPVAT from a regular cell size to an irregular size (Figure 2E). Compared with the saline group, the Ang II-infused mice showed a substantial decrease in adipocyte size in tPVAT (Figure 2E).

### 3.2. Molecular Changes during tPVAT Pathogenesis Analyzed by LC-MS/MS

To elucidate the molecular events occurring in tPVAT during Ang II infusion, a quantitative proteomic analysis, based on TMT labeling, was executed in the in vivo Ang II infusion model. A total of 30,079 peptide fragments, of which 27,160 were unique peptides corresponding to a total of 5037 proteins (Figure S1), were used. We received a good quality deviation during the data acquisition process using a high-quality Q Exactive mass spectrometer. The mass deviations of all the identified peptides were primarily distributed within 10 ppm, indicating that the identification results were accurate and reliable (Figure S2A). We obtained an ideal score with a median of 27.49, and more than 65.64% of the peptides scored higher than 20 when evaluating each MS2 spectrogram (Figure S2B). The protein ratio distribution of the saline and the Ang II-infused group are shown in Figure S2C. A 1.2-fold change cut-off, with a *p*-value < 0.05, was used to indicate significant changes in the abundance of the differentially expressed proteins (DEPs) between the saline and Ang II-infused groups.

### 3.3. Identification of DEPs

**Figure 3:**
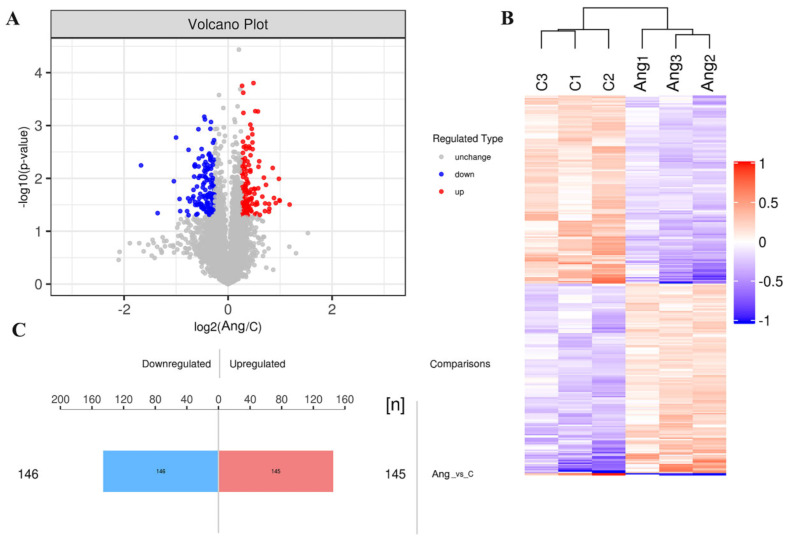
Differentially expressed proteins (DEPs) of tPVAT were identified in Ang II infused mice. (**A**) The volcano plot showed the distribution and number of DEPs between the saline and Ang II infused tPVAT. The red dots represent the up-regulated proteins, while the blue dots represent the down-regulated proteins. (**B**) Hierarchical clustering of DEPs from the Ang II group, compared with the saline group (three biological replicates per group). Red indicates upregulation and blue represents downregulation. (**C**) The number of DEPs. There are 145 up-regulated proteins and 146 down-regulated proteins.

In our study, the changes in expression of the DEPs between the saline and Ang II-infused groups were determined using a volcano plot and K-means clustering heatmaps as shown in Figure 3A,B. A total of 291 proteins were found to be significantly differentially expressed between the two groups, of which 145 proteins were upregulated and 146 proteins were downregulated (Figure 3C), indicating a drastic phenotypic alteration of tPVAT during Ang II infusion, when compared with the saline-infused group. The abbreviated lists of the up- and down-regulated proteins are provided in Table 1 and Table 2. As demonstrated in Table 1, most of the top 10 up-regulated proteins have been shown to be involved in epigenetic modification, cell phenotype changes, and differentiation. These proteins include KDM1A, PKN1, and ENGase [18,19,20]. In addition, CtBP1 participates in BAT differentiation [21], and TMEM41B is a novel ER-localized regulator of lipid mobilization and lipid droplets [22]. Regarding the down-regulated proteins, as demonstrated in Table 2, many of the top 10 proteins have been shown to participate in lipid metabolism, brown adipogenesis, and various pathological processes, such as coronary heart disease (CHD) and robust adiposity [23,24,25,26,27,28].

**Figure 4:**
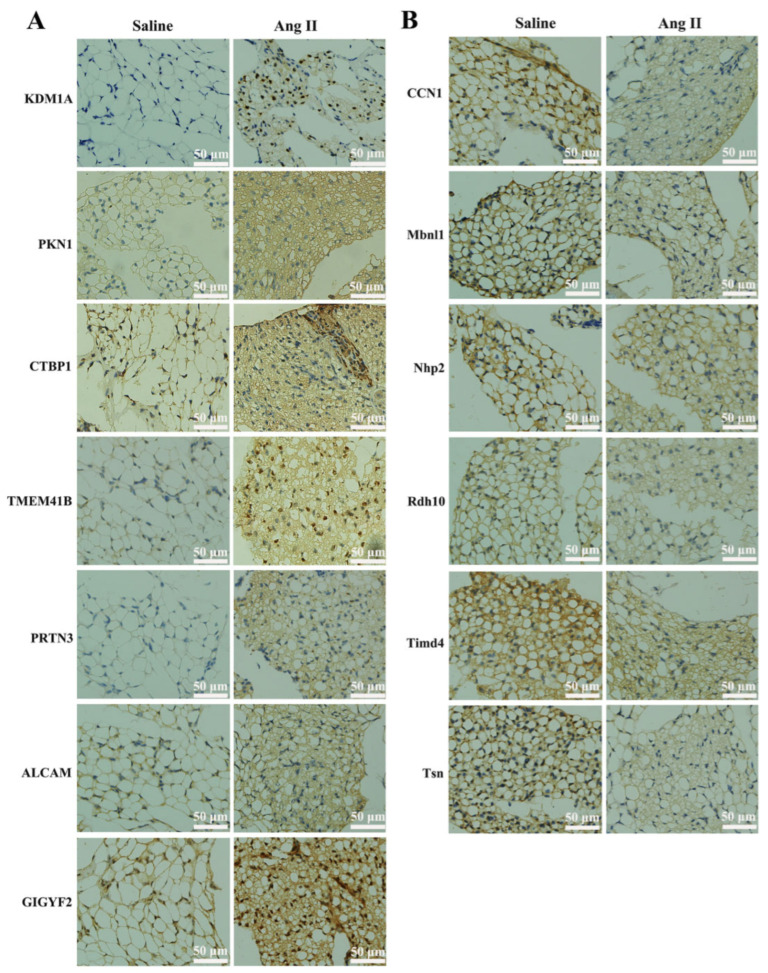
Immunohistochemical verification of DEPs. (**A**) Immunohistochemistry staining of up-regulated proteins, such as lysine-specific histone demethylase 1A (KDM1A), serine/threonine-protein kinase N1 (PKN1), C-terminal-binding protein 1 (CtBP1), transmembrane protein 41B (TMEM41B), myeloblastin (PRTN3), CD166 antigen (ALCAM), and GRB10-interacting GYF protein 2 (GIGYF2). Positive staining was indicated by brown coloration, and nuclei were stained with hematoxylin in blue. (**B**) Immunohistochemistry staining of down-regulated proteins, such as CCN family member 1 (CCN1), muscleblind-like protein 1 (MBNL1), H/ACA ribonucleoprotein complex subunit 2 (NHP2), retinol dehydrogenase 10 (RDH10), T-cell immunoglobulin and mucin domain-containing protein 4 (TIMD4), and Translin (TSN). Positive cells are indicated by brown coloration.

In addition, in order to verify the results of the TMT proteomics, we selected the top 10 up- or down-regulated proteins for immunohistochemical analyses. Figure 4 shows that the expressions of KDM1A, PKN1, CtBP1, TMEM41B, PRTN3, GIGYF2, and ALCAM were significantly up-regulated, while that of CCN1, MBNL1, NHP2, RDH10, TIMD4, and TSN were significantly decreased after Ang II infusion. The immunohistochemical results confirmed the expression pattern observed in the quantitative proteomics analysis. Since there were no suitable antibodies for ENGase, RasGRF2, ABHD4, Pate4, Selenof, Reps1, and COQ10B, we did not perform immunohistochemical verification of these proteins.

### 3.4. Bioinformatics Analysis of the Altered Proteins during Ang II Infusion

#### 3.4.1. GO Functional Annotation and Analysis

**Figure 5:**
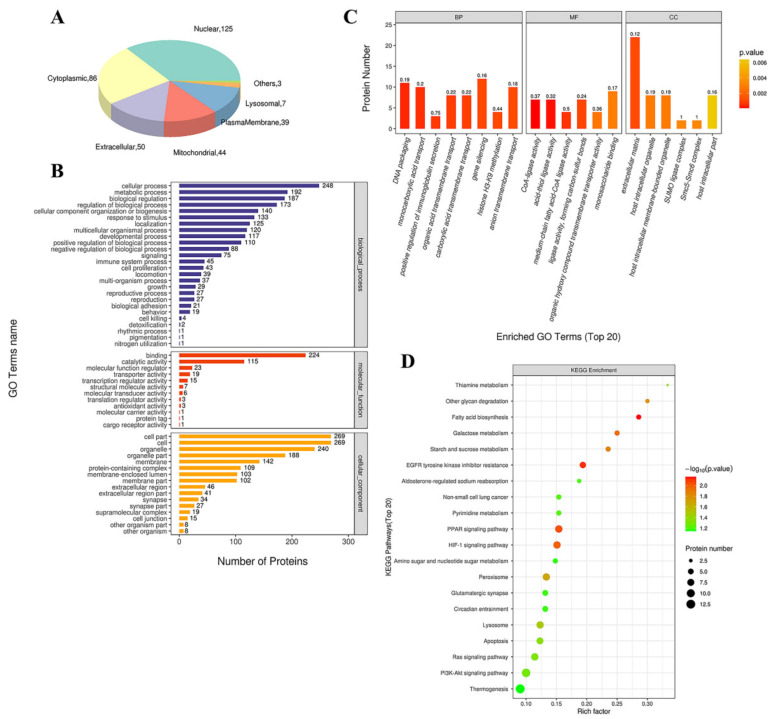
Functional analysis of DEPs. (**A**) Subcellular location of DEPs between saline and Ang II group. The numbers show the proteins located in different subcellular components. (**B**) Overall enriched GO terms. The vertical axis with different colors of the bar charts represents the significance of enrichment of the three categories (biological processes, molecular functions, cellular components). The horizontal axes represent the number of DEPs. (**C**) Top 20 enriched GO analyses of Ang II-altered proteins. BP: biological processes; MF: molecular functions; CC: cellular components. The color gradient from orange to red represents the *p* value. The numbers above the bar charts represent the richFactor (richFactor ≤ 1). (**D**) KEGG pathway enrichment bubble chart. The horizontal axes represent the richFactor (richFactor ≤ 1), which represents the ratio of the number of DEPs annotated to the KEGG pathway to the number of all identified proteins annotated to the KEGG category. The vertical axis represents the statistical results of DEPs under Top 20 KEGG pathways. The color of the bubble represents the significance of the enriched KEGG pathways. The color gradient from green to red represents the *p* value; the closer to red color, the lower the *p* value and the higher the significance level corresponding to the enrichment.

We used the subcellular structure prediction software CELLO to analyze the subcellular location of all the DEPs. As shown in Figure 5A, most of the proteins were distributed in the nucleus, cytoplasm, extracellular matrix, and mitochondria. The DEPs were then analyzed against the GO database using three sets of ontologies: biological process (BP), molecular function (MF), and cellular component (CC). The most enriched GO terms of BP, MF, and CC were annotated as a gene silencing (12 proteins, richFactor = 0.16), monosaccharide binding (9 proteins, richFactor = 0.17), extracellular matrix (22 proteins, richFactor = 0.12), respectively (Figure 5B,C and Table 3). Other important BPs were included in DNA packaging, monocarboxylic acid transport, positive regulation of immunoglobulin secretion, organic acid transmembrane transport, carboxylic acid transmembrane transport, etc. (Figure 5C). Other important MFs included CoA-ligase activity, acid-thiol ligase activity, medium-chain fatty acid-CoA ligase activity, ligase activity, forming carbon-sulfur bonds, and organic hydroxy compound transmembrane transporter activity (Figure 5C). Other important CCs included host intracellular organelles, host intracellular membrane-bounded organelles, SUMO ligase complex, and Smc5–Smc6 complex (Figure 5C).

#### 3.4.2. KEGG Pathway Analysis

By searching the major biological pathways and relevant regulatory processes involved in the KEGG, we analyzed all of the DEPs in the saline and Ang II infusion groups. The results of the KEGG pathway analysis showed that the significant enrichment pathways included fatty acid (FA) biosynthesis (4 proteins, richFactor = 0.29), EGFR tyrosine kinase inhibitor resistance (6 proteins, richFactor = 0.19), PPAR signaling pathway (8 proteins, richFactor = 0.15), HIF-1 signaling pathway (8 proteins, richFactor = 0.15), galactose metabolism (4 proteins, richFactor = 0.25), starch and sucrose metabolism (4 proteins, richFactor = 0.24), other glycan degradation (3 proteins, richFactor = 0.30), peroxisome (8 proteins, richFactor = 0.13), lysosome (8 proteins, richFactor = 0.12), thiamine metabolism (2 proteins, richFactor = 0.33), apoptosis (7 proteins, richFactor = 0.12), and Ras signaling pathway (8 proteins, richFactor = 0.11) (Figure 5D and Table 3).

**Figure 6:**
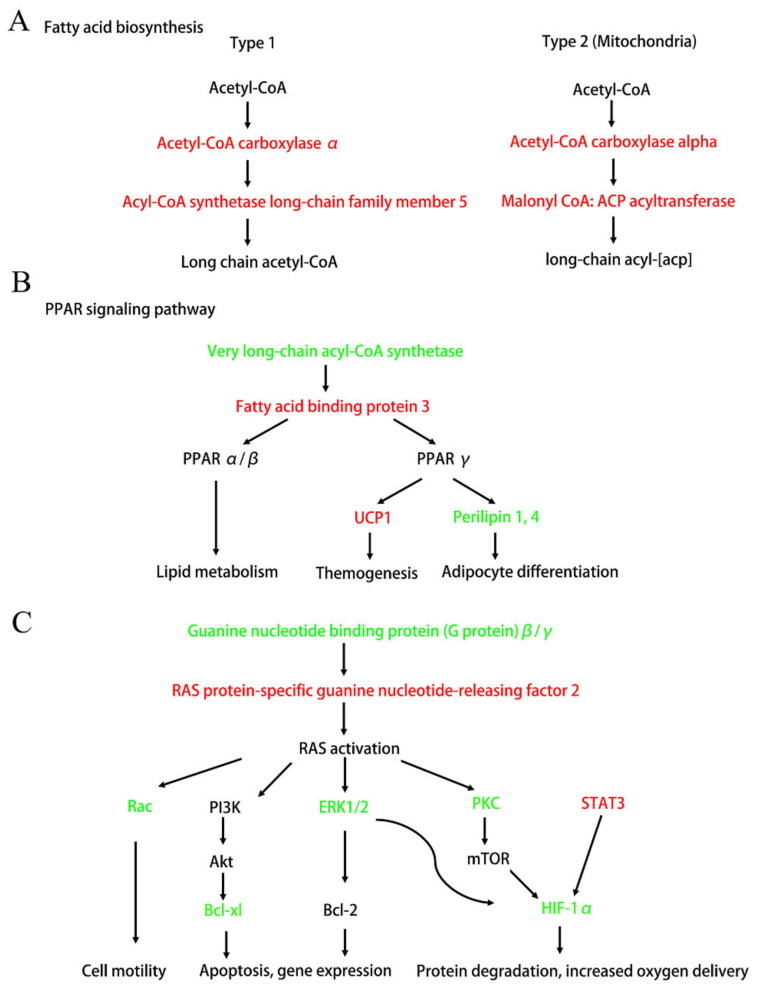
Diagram of the important signaling pathways that have changed after Ang II infusion. Up-regulated DEPs after Ang II infusion are shown in red. Down-regulated DEPs after Ang II infusion are shown in green. (**A**) Fatty acid biosynthetic pathway (simplified from Figure S3). (**B**) PPAR signaling pathway that affects lipid metabolism, themogenesis, and adipocyte differentiation (simplified from Figure S4). (**C**) Ras and its downstream signaling pathways (simplified from Figures S5–S7). Ras-Rac signaling affects cell motility. Ras-PI3K-Akt and Ras-ERK1/2 signaling affects cell apoptosis and gene expression. Ras-HIF1α pathway affects cell protein degradation and oxygen delivery.

Next, we connected these significant enrichment pathways and found that Ang II infusion deeply affected the fat metabolism and adipocyte differentiation (Figure 6A,B). Figure 6A shows that the up-regulation of acetyl-CoA carboxylase α and acyl-CoA synthetase long-chain family member 5 significantly affected the synthesis of long-chain FAs. In addition, Figure 6B shows that Ang II infusion affected the PPAR signaling pathway by regulating very long-chain acyl-CoA synthase (ACSVL) and FA binding protein 3 (FABP3), and thereby affected lipid metabolism, thermogenesis, and adipocyte differentiation. Additionally, we also found that Ang II infusion could affect many downstream signaling pathways, such as Rac, PI3K/Akt, MAPK, and the HIF-1α pathways, by regulating the Ras signaling that, in turn, affected many cell functions and participated in pathogenesis (Figure 6C).

#### 3.4.3. PPI Analysis

**Figure 7:**
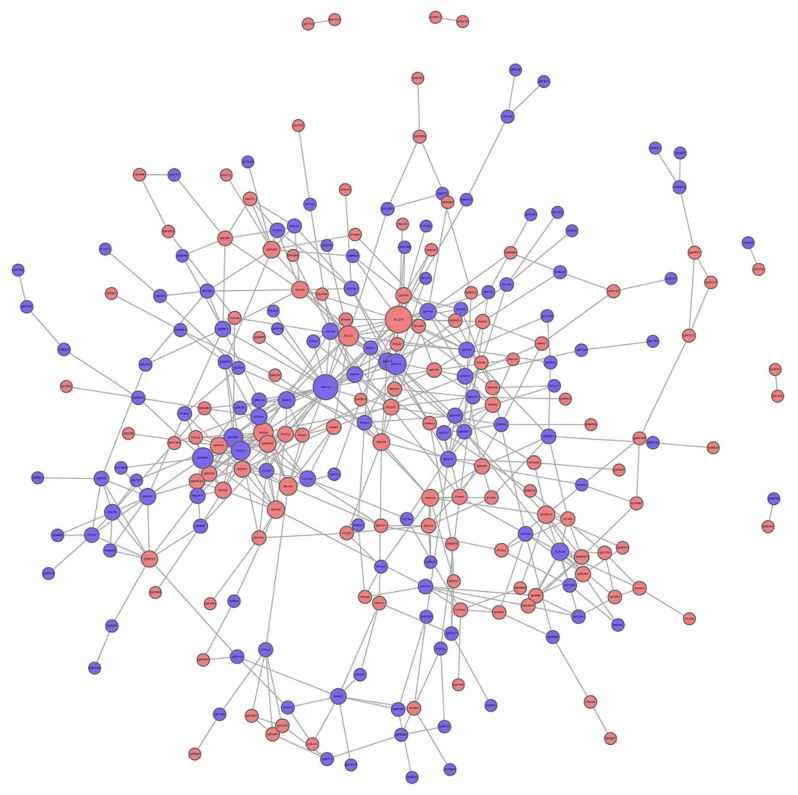
Protein protein interaction (PPI) analysis of DEPs between saline and Ang II infusion group. The circled nodes represent DEPs, and the lines represent protein-protein interactions. Red circles represent up-regulation proteins. Blue circles represent down-regulation proteins. The size of the circle indicates the degree of protein connectivity.

In the PPI network, there were 20 high-connectivity degree DEPs with a large circle and a degree value of more than 10 between the saline and Ang II groups (Figure 7). These high-connectivity DEPs were identified as fibronectin (P11276), actin (P60710), mitogen-activated protein kinase (MAPK) 3 (Q63844), H/ACA ribonucleoprotein complex subunit 2 (Q9CRB2), signal transducer and activator of transcription (STAT) 3 (P42227), proliferation-associated protein 2G4 (P50580), nucleolar GTP-binding protein 1 (Q99ME9), eukaryotic translation initiation factor 6 (O55135), COP9 signalosome complex subunit 2 (P61202), Bcl-2-like protein 1 (Q64373), ACSVL (O35488), acetyl-CoA carboxylase 1 (Q5SWU9), 60S ribosomal protein L22 (P67984), hexokinase-2 (O08528), superoxide dismutase [Cu-Zn] (P08228), eukaryotic translation initiation factor 4E (P63073), high mobility group protein B1 (P63158), eukaryotic translation initiation factor 2 subunit 2 (Q99L45), lysine-specific histone demethylase 1A (Q6ZQ88), and ubiquitin carboxyl-terminal hydrolase isozyme L5 (Q9WUP7) (Table 4). Some high-connectivity node proteins in Table 4 were related to the MAPK-Erk, STAT, FA biosynthesis, PPAR, and apoptosis signaling pathways, and these were highly consistent with those obtained using KEGG.

## 4. Discussion

PVAT is the connective tissue surrounding most of the systemic blood vessels and is now considered to be an important endocrine tissue that maintains the vascular homeostasis. Healthy PVAT has anti-contraction, anti-inflammation, and anti-oxidation effects [6]. According to the region of the vascular layer, where PVAT is located, PVAT shows phenotypic and functional heterogeneity [29]. For example, in the aorta of rodents, PVAT is primarily composed of WAT in the abdominal region, while PVAT is primarily composed of BAT in the thoracic region [30]. Angiotensinogen (AGT) was first found in BAT around the aorta in 1987 [31]. Evidence has demonstrated that the local renin-angiotensin system (RAS) may play an important role in adipocyte growth and differentiation [32]. However, the effect of pathological local RAS activation on the normal physiological function of PVAT is still unknown. Because Ang II is the primary component of RAS, we used the mice Ang II infusion model in the present study to simulate the pathological increase of local RAS at tPVAT. The results showed that Ang II infusion significantly increased the body weight and tPVAT weight of mice. In addition, the morphology of tPVAT also changed, and the distribution of tPVAT around the thoracic aorta appeared uneven (Figure 2A–D). To further verify the influence of Ang II infusion on the structure of tPVAT, we performed H&E staining on tPVAT tissue. The result showed that Ang II significantly changed the diameter and size of adipocytes in tPVAT (Figure 2E). These results indicate that the pathological activation of local RAS may greatly change the structure and function of tPVAT, and thereby affect vascular function.

To further clarify the molecular mechanisms of tPVAT structural changes caused by Ang II infusion, TMT label-based nanoscale LC–MS/MS was used in the present study. We identified a number of novel proteins associated with Ang II infusion and extended our understanding of this process. A total of 291 with significantly differential expression (145 up-regulation and 146 down-regulation) were identified in three biological replicates, and the hierarchical cluster analysis showed that these proteins were wel-distinguished. This indicated that the overall protein screening had reasonable accuracy. Additionally, we selected the top 10 differential expressed proteins with appropriate antibodies for immunohistochemical verification, and the data showed trends that paralleled those observed in the TMT results (Figure 4). To our knowledge, this is the first and most comprehensive large-scale proteomic profiling of Ang II-infused tPVAT, and the data will facilitate future studies of pathological tPVAT after the local activation of RAS.

The GO enrichment results indicated that the most enriched BP, MF, and CC categories after Ang II infusion were gene silencing (up regulation: *CtBP1*, *KDM1A*, *GIGYF2*, *MRPL44*, *STAT3*, *H3C1*; down regulation: *BAZ2A*, *MECP2*, *TDRKH*, *H1-2*, *H1.5*, *eIF6*), monosaccharide binding (up regulation: *HK2*, *P4HA1*, *OGFOD3*, *PFKL*, *GPI*; down regulation: *Mbl2*, *MANBA*, *DBH*, *GNPNAT1*), and extracellular matrix (up regulation: *FN1*, *POSTN*, *CTSL*, *TNC*, *MATN2*, *TGFBI*, *ITIH3*, *SRPX2*, *HSD17B12*, *ALPL*, *LRRc17*; down regulation: *ANXA2*, *FBLN5*, *ANXA4*, *LTBP-4*, *ANXA11*, *AGRN*, *MFAP1a*, *Mbl2*, *EFEMP1*, *CCN1*, *Marco*), respectively. We found that the genes in gene silencing, such as *CtBP1*, *KDM1A*, *MECP2*, *STAT3*, *eIF6*, were involved in regulating adipocyte inflammatory, differentiation, reconstruction, oxidation, and metabolism [21,33,34,35,36,37,38]. In addition, a large number of extracellular matrix components and regulatory proteins have changed after Ang II infusion, and these may participate in regulating cell proliferation, migration, and other phenotypic transformations.

As highlighted by the KEGG analysis, the most important networks, after Ang II infusion, were related to FA biosynthesis, PPAR signaling, Ras signaling, MAPK-Erk pathway, STAT3, and the HIF-1α pathway (Figure 6). Although it has been reported that Ang II induces adipocyte dysfunction, the mechanisms of this action are not very clear [39]. Our current study found that Ang II affected the synthesis of long-chain FAs, by up-regulating acyl-CoA carboxylase α and down-regulating ACSVL (Figure 6A,B). Additionally, these two FA synthesis-related enzymes had a high connectivity degree in the PPI analysis (Figure 7, Table 4) and could serve as possible novel molecular targets for Ang II action. Furthermore, Ang II affected the PPARγ signaling by up-regulating FABP3, which, in turn, promoted UCP1 and suppressed perilipin1, 4 expressions (Figure 6B). FABP3 is one of the members of the lipid-binding proteins superfamily. It is both membrane-bound, aiding cellular long-chain FA uptake, and cytoplasmic, being crucial to intracellular transport of FAs to sites of metabolic conversion [40]. It has been reported that the FABP3-PPARα axis has an indispensable role in Ang II-induced cardiac hypertrophy and heart failure [41]. Therefore, we speculate that the FABP3-PPARα axis also plays an important role in Ang II-induced tPVAT pathogenesis. In addition, Ang II promoted UCP1 and inhibited perilipin 4 expressions (Figure 6B). UCP1 primarily exists in BAT and is a mitochondrial protein embedded in the inner membrane of mitochondria, and it can dissipate the energy stored in mitochondrial electrochemical gradient as heat and “decouple” from ATP synthesis [42]. The thermogenesis of BAT and the energy steady state of the system depend entirely on the action of UCP1, and UCP1 up-regulation indicates adipocyte browning [43,44]. Therefore, the pathological increase of Ang II in this study may affect tPVAT thermometabolism and preadipocyte browning by up-regulating UCP1, which in turn affects vascular function. Perilipin proteins were discovered in the adipocyte, where they regulate lipid storage and lipolysis and are considered direct PPARγ targets [45,46]. Perilipin 1 appears to be a highly specific marker for adipocytic differentiation [47]. Perilipin 4 participates in the formation of lipid droplets and is an adipocyte regulator of triglyceride synthesis and packaging [48]. In the current study, Ang II may regulate the differentiation and function of adipocytes at mice tPVAT by down-regulating perilipin 1 and 4. In brief, the present study suggests that UCP1 and perilipin may be new targets of Ang II infusion, allowing Ang II to directly participate in the regulation of lipid metabolism, transportation, and adipocyte differentiation.

The Ras-ERK pathway is largely known for its regulation of cell proliferation, differentiation, survival, and drug-mediated behaviors. A recent study found that RasGRF2 (RAS protein-specific guanine nucleotide-releasing factor 2) mediates cocaine self-administration (SA) in mice via an ERK-dependent mechanism [49]. Our study discovered that RasGRF2 was up-regulated after Ang II infusion and could serve as a novel target for Ang II action, and the RasGRF2-Ras-ERK signal axis may play an important role in Ang II-mediated tPVAT pathogenesis. In addition, hypoxia inducible factor 1α (HIF-1α) has been reported to participate in the Ang II-mediated inflammatory response process [50], cardiomyocyte hypertrophy [51], preautonomic neurons activation [52], and vascular remodeling [53]. Our study showed a decreased expression level of HIF-1α after Ang II infusion and indicated that HIF-1α may be also a novel biomarker for Ang II-mediated tPVAT pathogenesis. In addition, RasGRF2-ERK-HIF-1α, RasGRF2-PKC-HIF-1α, and the STAT3-HIF-1α axis may be three important pathways at tPVAT with locally pathological activation of RAS, which can be further studied by subsequent researchers. A recent report showed that STAT3/HIF-1α signaling participates in peritoneal fibrosis during long-term peritoneal dialysis (PD) treatment [54]. Our results indicated an increased expression of STAT3 after Ang II infusion, and STAT3 had a high connectivity degree in the PPI analysis (Figure 6C and Table 4). Thus, STAT3 signaling may play an important role in Ang II-mediated phenotypic changes of tPVAT.

In conclusion, the present quantitative proteomic study provided the most comprehensive proteome profiles of mice tPVAT and a list of DEPs between saline-infused and Ang II-infused tPVAT. The bioinformatics analyses found that the functions of these altered proteins were primarily concentrated in gene silencing and the extracellular matrix. More importantly, the present study provided novel molecular targets of Ang II, such as acyl-CoA carboxylase α, ACSVL, UCP1, perilipin, and RasGRF2, as well as important signaling pathways, such as FA biosynthesis, FABP3-PPARα/γ, RasGRF2-ERK-HIF-1α, RasGRF2-PKC-HIF-1α, and STAT3-HIF-1α, during Ang II-induced tPVAT pathogenesis. The data provided here will promote our understanding of the effects of local RAS activation on PVAT and vascular function.

## Figures and Tables

**Table 1:** List of up-regulated proteins during Ang II infusion.

Accession NO.	Gene Name	Protein Name	Accession NO.	Gene Name	Protein Name
**Q6ZQ88**	***KDM1A***	**Lysine-specific histone demethylase 1A**	Q9CPV3	*MRPL42*	39S ribosomal protein L42, mitochondrial
**P70268**	***PKN1***	**Serine/threonine-protein kinase N1**	Q8VCW8	*ACSF2*	Medium-chain acyl-CoA ligase ACSF2, mitochondrial
**Q8BX80**	***ENGase***	**Cytosolic endo-beta-N-acetylglucosaminidase**	Q9D0F9	*PGM1*	Phosphoglucomutase-1
**O88712**	***CtBP1***	**C-terminal-binding protein 1**	P53986	*SLC16A1*	Monocarboxylate transporter 1
**Q8K1A5**	***Tmem41b***	**Transmembrane protein 41B**	Q8BGQ1	*VIPAS39*	Spermatogenesis-defective protein 39 homolog
**P70392**	***RasGRF2***	**Ras-specific guanine nucleotide-releasing factor 2**	Q9D2R0	*AACS*	Acetoacetyl-CoA synthetase
**Q61096**	***PRTN3***	**Myeloblastin**	Q9CXR1	*DHRS7*	Dehydrogenase/reductase SDR family member 7
**Q8VD66**	***ABHD4***	**(Lyso)-N-acylphosphatidylethanolamine lipase**	Q8K3K7	*AGPAT2*	1-acyl-sn-glycerol-3-phosphate acyltransferase beta
**Q6Y7W8**	***GIGYF2***	**GRB10-interacting GYF protein 2**	Q924N4	*SLC12A6*	Solute carrier family 12 member 6
**Q61490**	***ALCAM***	**CD166 antigen**	Q922Z0	*DDO*	D-aspartate oxidase
Q8CHD8	*RAB11FIP3*	Rab11-family interacting protein 3	Q14DH7	*ACSS3*	Acyl-CoA synthetase short-chain family member 3, mitochondrial
Q8R054	*SRPX2*	Sushi repeat-containing protein SRPX2	Q00898	*SERPINA1E*	Alpha-1-antitrypsin 1–5
Q3UVG3	*FAM91A1*	Protein FAM91A1	Q9JJF9	*SPPL2a*	Signal peptide peptidase-like 2A
O89017	*LGMN*	Legumain	Q9CS72	*FILIP1*	Filamin-A-interacting protein 1
Q8R1R3	*StarD7*	StAR-related lipid transfer protein 7, mitochondrial	Q9QXG4	*ACSS2*	Acetyl-coenzyme A synthetase, cytoplasmic
Q924W5	*Smc6*	Structural maintenance of chromosomes protein 6	P28063	*PSMB8*	Proteasome subunit beta type-8
Q64516	*GK*	Glycerol kinase	Q5RKZ7	*MOCS1*	Molybdenum cofactor biosynthesis protein 1
Q9D023	*MPC2*	Mitochondrial pyruvate carrier 2	P67984	*Rpl22*	60S ribosomal protein L22
E9Q1P8	*IRF2BP2*	Interferon regulatory factor 2-binding protein 2	Q99KF1	*TMED9*	Transmembrane emp24 domain-containing protein 9
P12242	*UPC1*	Mitochondrial brown fat uncoupling protein 1	Q9CY73	*MRPL44*	39S ribosomal protein L44, mitochondrial
P11404	*FABP3*	Fatty acid-binding protein, heart	Q8BHJ6	*SERINC5*	Serine incorporator 5
P09470	*ACE*	Angiotensin-converting enzyme	P70699	*GAA*	Lysosomal alpha-glucosidase
Q8BMB3	*eIF4E2*	Eukaryotic translation initiation factor 4E type 2	Q3U4G3	*XXYLT1*	Xyloside xylosyltransferase 1
Q9D136	*OGFOD3*	2-oxoglutarate and iron-dependent oxygenase domain-containing protein 3	Q8C7H1	*MMAA*	Methylmalonic aciduria type A homolog, mitochondrial
Q62009	*POSTN*	Periostin	Q80VW7	*AKNA*	Microtubule organization protein AKNA
Q78JN3	*Eci3*	Enoyl-CoA delta isomerase 3, peroxisomal	O54940	*BNIP2*	BCL2/adenovirus E1B 19 kDa protein-interacting protein 2
P68433	*H3C1*	Histone H3.1	Q6PEE2	*CTIF*	CBP80/20-dependent translation initiation factor
Q5SF07	*IGF2BP2*	Insulin-like growth factor 2 mRNA-binding protein 2	Q9D4H1	*EXOC2*	Exocyst complex component 2
Q9JM90	*STAP1*	Signal-transducing adaptor protein 1	Q8CFV9	*RFK*	Riboflavin kinase
Q80YX1	*TNC*	Tenascin	P29595	*NEDD8*	NEDD8
Q9DCE5	*PAK1IP1*	p21-activated protein kinase-interacting protein 1	Q9Z0P5	*TWF2*	Twinfilin-2
Q8JZR0	*ACSL5*	Long-chain-fatty-acid--CoA ligase 5	Q9CXJ1	*EARS2*	Probable glutamate--tRNA ligase, mitochondrial
Q9CXD9	*LRRc17*	Leucine-rich repeat-containing protein 17	Q8VHQ9	*ACOT11*	Acyl-coenzyme A thioesterase 11
Q8BWJ3	*PHKA2*	Phosphorylase b kinase regulatory subunit alpha, liver isoform	Q920B9	*SUPT16H*	FACT complex subunit SPT16
Q811L6	*MAST4*	Microtubule-associated serine/threonine-protein kinase 4	Q9DCV4	*RMDN1*	Regulator of microtubule dynamics protein 1
P61202	*COPS2*	COP9 signalosome complex subunit 2	P06745	*GPI*	Glucose-6-phosphate isomerase
Q1W617	*SHROOM4*	Protein SHROOM4	O08600	*EndoG*	Endonuclease G, mitochondrial
P63158	*HMGB1*	High mobility group protein B1	O08746	*MATN2*	Matrilin-2
P36536	*SAR1A*	GTP-binding protein SAR1A	Q78J03	*MsrB2*	Methionine-R-sulfoxide reductase B2, mitochondrial
Q8BJ03	*COX15*	Cytochrome c oxidase assembly protein COX15 homolog	Q99L45	*EIF2S2*	Eukaryotic translation initiation factor 2 subunit 2
Q8BHC0	*LYVE-1*	Lymphatic vessel endothelial hyaluronic acid receptor 1	P97452	*BOP1*	Ribosome biogenesis protein BOP1
Q8VE11	*MTMR6*	Myotubularin-related protein 6	O08528	*HK2*	Hexokinase-2
Q91V76	*C11orf54*	Ester hydrolase C11orf54 homolog	Q9Z1T1	*Ap3B1*	AP-3 complex subunit beta-1
Q64008	*Rab34*	Ras-related protein Rab-34	Q920A5	*SCPEP1*	Retinoid-inducible serine carboxypeptidase
P30993	*C5aR1*	C5a anaphylatoxin chemotactic receptor 1	Q91VU0	*FAM3C*	Protein FAM3C
Q91WC3	*ACSL6*	Long-chain-fatty-acid--CoA ligase 6	Q9CQE7	*ERGIC3*	Endoplasmic reticulum-Golgi intermediate compartment protein 3
Q7TPM3	*TRIM17*	E3 ubiquitin-protein ligase TRIM17	P50580	*PA2G4*	Proliferation-associated protein 2G4
P70122	*SBDS*	Ribosome maturation protein SBDS	Q9CZD5	*MTIF3*	Translation initiation factor IF-3, mitochondrial
Q8CHG3	*GCC2*	GRIP and coiled-coil domain-containing protein 2	P01631		Ig kappa chain V-II region 26–10
Q80X95	*RRAGA*	Ras-related GTP-binding protein A	Q9D125	*MRPS25*	28S ribosomal protein S25, mitochondrial
Q8R3F5	*MCAT*	Malonyl-CoA-acyl carrier protein transacylase, mitochondrial	Q5SW19	*CLUH*	Clustered mitochondria protein homolog
Q99LJ6	*GPX7*	Glutathione peroxidase 7	Q7TPE5	*SLC7A6OS*	Probable RNA polymerase II nuclear localization protein SLC7A6OS
O70503	*HSD17B12*	Very-long-chain 3-oxoacyl-CoA reductase	P42227	*STAT3*	Signal transducer and activator of transcription 3
P12382	*PFKL*	ATP-dependent 6-phosphofructokinase, liver type	Q9Z0S1	*BPNT-1*	3′(2′),5′-bisphosphate nucleotidase 1
Q61704	*ITIH3*	Inter-alpha-trypsin inhibitor heavy chain H3	P09242	*ALPL*	Alkaline phosphatase, tissue-nonspecific isozyme
P06797	*CTSL*	Cathepsin L1	P12265	*GUSB*	Beta-glucuronidase
Q80WG5	*LRRC8A*	Volume-regulated anion channel subunit LRRC8A	Q61391	*MME*	Neprilysin
Q9CQV4	*RETREG3*	Reticulophagy regulator 3	P82198	*TGFBI*	Transforming growth factor-beta-induced protein ig-h3
Q8BGR6	*ARL15*	ADP-ribosylation factor-like protein 15	Q9WUP7	*UCHL5*	Ubiquitin carboxyl-terminal hydrolase isozyme L5
P11276	*FN1*	Fibronectin	P52196	*TST*	Thiosulfate sulfurtransferase
P70460	*VASP*	Vasodilator-stimulated phosphoprotein	Q9QZA0	*CA5B*	Carbonic anhydrase 5B, mitochondrial
P06339	*H2-T23*	H-2 class I histocompatibility antigen, D-37 alpha chain	Q8QZY9	*SF3B4*	Splicing factor 3B subunit 4
Q65Z40	*Wapl*	Wings apart-like protein homolog	P34914	*EPHX2*	Bifunctional epoxide hydrolase 2
P10126	*Eef1a1*	Elongation factor 1-alpha 1	O54946	*DNAJB6*	DnaJ homolog subfamily B member 6
P54823	*Ddx6*	Probable ATP-dependent RNA helicase DDX6	Q9CQ86	*MIEN1*	Migration and invasion enhancer 1
Q8BUE4	*Aifm2*	Apoptosis-inducing factor 2	Q8CG46	*SMC5*	Structural maintenance of chromosomes protein 5
O35143	*ATP5IF1*	ATPase inhibitor, mitochondrial	Q60715	*P4HA1*	Prolyl 4-hydroxylase subunit alpha-1
Q9Z0V8	*Timm17a*	Mitochondrial import inner membrane translocase subunit Tim17-A	P15105	*GLUL*	Glutamine synthetase
Q9CWX9	*Ddx47*	Probable ATP-dependent RNA helicase DDX47	P08228	*SOD1*	Superoxide dismutase [Cu-Zn]
O35344	*Kpna3*	Importin subunit alpha-4	Q5SWU9	*ACACA*	Acetyl-CoA carboxylase 1
O54950	*Prkag1*	5’-AMP-activated protein kinase subunit gamma-1	Q91Z96	*BMP2K*	BMP-2-inducible protein kinase
Q99P65	*Slc29a3*	Equilibrative nucleoside transporter 3	Q9CYK1	*WARS2*	Tryptophan--tRNA ligase, mitochondrial
O09111	*Ndufb11*	NADH dehydrogenase [ubiquinone] 1 beta subcomplex subunit 11, mitochondrial			

This table contains the 145 proteins that displayed more than 1.2-fold up-regulation in Ang II-infused mice in three independent experiments. The protein accession number, gene name, and name of each protein are provided here. The proteins are listed in descending order, according to their fold change (the fold change can be seen in Table S2), and the top 10 up-regulated proteins appear in bold.

**Table 2:** List of down-regulated proteins during Ang II infusion.

Accession NO.	Gene Name	Protein Name	Accession NO.	Gene Name	Protein Name
**P18406**	***CCN1***	**CCN family member 1**	A2RTL5	*RSRC2*	Arginine/serine-rich coiled-coil protein 2
**Q09098**	***Pate4***	**Prostate and testis expressed protein 4**	Q8BL86	*MBLAC2*	Metallo-beta-lactamase domain-containing protein 2
**Q9ERR7**	***Selenof***	**Selenoprotein F**	Q6ZWY8	*TMSB10*	Thymosin beta-10
**O54916**	***Reps1***	**RalBP1-associated Eps domain-containing protein 1**	Q9D5V6	*Syap1*	Synapse-associated protein 1
**Q9CRB2**	***NHP2***	**H/ACA ribonucleoprotein complex subunit 2**	Q8BK08	*TMEM11*	Transmembrane protein 11, mitochondrial
**Q6U7R4**	***TIMD4***	**T-cell immunoglobulin and mucin domain-containing protein 4**	Q80X80	*C2CD2L*	Phospholipid transfer protein C2CD2L
**Q9JKP5**	***MBNL1***	**Muscleblind-like protein 1**	Q9WV85	*NME3*	Nucleoside diphosphate kinase 3
**Q62348**	***TSN***	**Translin**	Q9DCL2	*CIAO2A*	Cytosolic iron-sulfur assembly component 2A
**Q8VCH7**	***RDH10***	**Retinol dehydrogenase 10**	Q61285	*ABCD2*	ATP-binding cassette sub-family D member 2
**Q3THF9**	***COQ10B***	**Coenzyme Q-binding protein COQ10 homolog B, mitochondrial**	P70333	*HNRNPH2*	Heterogeneous nuclear ribonucleoprotein H2
Q9JK38	*GNPNAT1*	Glucosamine 6-phosphate N-acetyltransferase	Q69ZP3	*PNKD*	Probable hydrolase PNKD
Q05144	*Rac2*	Ras-related C3 botulinum toxin substrate 2	O35864	*COPS5*	COP9 signalosome complex subunit 5
P60710	*ACTB*	Actin, cytoplasmic 1	Q61464	*ZNF638*	Zinc finger protein 638
Q9Z140	*CPNE6*	Copine-6	Q9JM14	*NT5C*	5’(3’)-deoxyribonucleotidase, cytosolic type
P47955	*RPLP1*	60S acidic ribosomal protein P1	Q9D1L0	*CHCHD2*	Coiled-coil-helix-coiled-coil-helix domain-containing protein 2
P63073	*eIF4E*	Eukaryotic translation initiation factor 4E	Q3TMW1	*CCDC102A*	Coiled-coil domain-containing protein 102A
Q8CH72	*TRIM32*	E3 ubiquitin-protein ligase TRIM32	Q9Z0V7	*TIMM17B*	Mitochondrial import inner membrane translocase subunit Tim17-B
P83870	*PHF5A*	PHD finger-like domain-containing protein 5A	O88895	*HDAC3*	Histone deacetylase 3
P41317	*MBL2*	Mannose-binding protein C	P97370	*ATP1B3*	Sodium/potassium-transporting ATPase subunit beta-3
P15864	*H1-2*	Histone H1.2	P26645	*MARCKS*	Myristoylated alanine-rich C-kinase substrate
Q91WU5	*AS3MT*	Arsenite methyltransferase	Q3TEA8	*HP1BP3*	Heterochromatin protein 1-binding protein 3
O54962	*BANF1*	Barrier-to-autointegration factor	Q9D1I2	*CARD19*	Caspase recruitment domain-containing protein 19
O70451	*SLC16A7*	Monocarboxylate transporter 2	Q3UEZ8	*SLC10A4*	Sodium/bile acid cotransporter 4
P26011	*ITGB7*	Integrin beta-7	Q99ME9	*GTPBP4*	Nucleolar GTP-binding protein 1
Q9JHH9	*COPZ2*	Coatomer subunit zeta-2	Q0VGM9	*RTEL1*	Regulator of telomere elongation helicase 1
O54788	*DFFB*	DNA fragmentation factor subunit beta	Q9JLQ0	*CD2AP*	CD2-associated protein
P82348	*SGCG*	Gamma-sarcoglycan	O70370	*CTSS*	Cathepsin S
B1AVZ0	*UPRT*	Uracil phosphoribosyltransferase homolog	Q69ZF3	*GBA2*	Non-lysosomal glucosylceramidase
Q9JHI7	*EXOSC9*	Exosome complex component RRP45	Q8K124	*PLEKHO2*	Pleckstrin homology domain-containing family O member 2
Q61207	*PSAP*	Prosaposin	P19973	*LSP1*	Lymphocyte-specific protein 1
O35638	*STAG2*	Cohesin subunit SA-2	P62874	*GNB1*	Guanine nucleotide-binding protein G(I)/G(S)/G(T) subunit beta-1
Q91YE5	*BAZ2A*	Bromodomain adjacent to zinc finger domain protein 2A	Q80VL1	*TDRKH*	Tudor and KH domain-containing protein
Q9CY57	*Chtop*	Chromatin target of PRMT1 protein	Q9Z2D6	*MECP2*	Methyl-CpG-binding protein 2
O55135	*eIF6*	Eukaryotic translation initiation factor 6	Q61503	*NT5E*	5’-nucleotidase
Q8R3B7	*BRD8*	Bromodomain-containing protein 8	Q8K2Q5	*CHCHD7*	Coiled-coil-helix-coiled-coil-helix domain-containing protein 7
Q8R143	*PTTG1IP*	Pituitary tumor-transforming gene 1 protein-interacting protein	Q8BP48	*MetAP1*	Methionine aminopeptidase 1
Q9D2V5	*AAR2*	Protein AAR2 homolog	Q64237	*DBH*	Dopamine beta-hydroxylase
P28798	*GRN*	Progranulin	O35226	*PSMD4*	26S proteasome non-ATPase regulatory subunit 4
Q9R0Y5	*AK-1*	Adenylate kinase isoenzyme 1	E9PZM4	*CHD2*	Chromodomain-helicase-DNA-binding protein 2
P03899	*MTND3*	NADH-ubiquinone oxidoreductase chain 3	P26369	*U2AF2*	Splicing factor U2AF 65 kDa subunit
Q8JZS0	*LIN7A*	Protein lin-7 homolog A	P56379	*ATP5MPL*	ATP synthase subunit ATP5MPL, mitochondrial
Q91V12	*ACOT7*	Cytosolic acyl coenzyme A thioester hydrolase	E9Q5C9	*NOLC1*	Nucleolar and coiled-body phosphoprotein 1
Q00724	*RBP4*	Retinol-binding protein 4	P59279	*RAB2B*	Ras-related protein Rab-2B
Q78HU7	*GYPC*	Glycophorin-C	Q8R332	*NUP58*	Nucleoporin p58/p45
Q9QZB0	*RGS17*	Regulator of G-protein signaling 17	P97384	*ANXA11*	Annexin A11
Q8BGX2	*TIMM29*	Mitochondrial import inner membrane translocase subunit Tim29	Q5XG73	*ACBD5*	Acyl-CoA-binding domain-containing protein 5
P97429	*ANXA4*	Annexin A4	Q60754	*MARCO*	Macrophage receptor MARCO
O55186	*CD59a*	CD59a glycoprotein	A2AJB2	*TMEM141*	Transmembrane protein 141
Q8BLV3	*SLC9A7*	Sodium/hydrogen exchanger 7	Q8K2I4	*MANBA*	Beta-mannosidase
Q0VBL1	*TIGD2*	Tigger transposable element-derived protein 2	P20444	*PRKCA*	Protein kinase C alpha type
O09117	*SYPL1*	Synaptophysin-like protein 1	P43276	*H1.5*	Histone H1.5
Q9WTR5	*CDH13*	Cadherin-13	Q8CGN5	*PLIN1*	Perilipin-1
Q8VCF0	*MAVS*	Mitochondrial antiviral-signaling protein	Q63844	*MAPK3*	Mitogen-activated protein kinase 3
O88492	*PLIN4*	Perilipin-4	Q3UMY5	*EML4*	Echinoderm microtubule-associated protein-like 4
Q80US4	*ACTR5*	Actin-related protein 5	Q6DIC0	*SNF2L2*	Probable global transcription activator SNF2L2
Q8C3X8	*LMF2*	Lipase maturation factor 2	P07356	*ANXA2*	Annexin A2
P99028	*UQCRH*	Cytochrome b-c1 complex subunit 6, mitochondrial	Q9WVH9	*FBLN5*	Fibulin-5
P58742	*AAAS*	Aladin	P43135	*NR2F2*	COUP transcription factor 2
Q6PCP5	*Mff*	Mitochondrial fission factor	Q8R4R6	*NUP35*	Nucleoporin NUP35
O35488	*SLC27A2*	Very long-chain acyl-CoA synthetase	P11798	*CAMK2A*	Calcium/calmodulin-dependent protein kinase type II subunit alpha
Q9DAS9	*GNG12*	Guanine nucleotide-binding protein G(I)/G(S)/G(O) subunit gamma-12	Q8BHL3	*TBC1D10B*	TBC1 domain family member 10B
Q9D1G5	*LRRC57*	Leucine-rich repeat-containing protein 57	Q8BTI8	*SRRNM2*	Serine/arginine repetitive matrix protein 2
Q8BGD8	*COA6*	Cytochrome c oxidase assembly factor 6 homolog	P97952	*SCN1B*	Sodium channel subunit beta-1
Q9CRB8	*MTFP1*	Mitochondrial fission process protein 1	P51912	*SLC1A5*	Neutral amino acid transporter B (0)
Q99MS7	*EHBP1L1*	EH domain-binding protein 1-like protein 1	Q8BPB5	*EFEMP1*	EGF-containing fibulin-like extracellular matrix protein 1
Q61029	*TMPO*	Lamina-associated polypeptide 2, isoforms beta/delta/epsilon/gamma	Q64373	*BCL2L1*	Bcl-2-like protein 1
Q91YU6	*LZTS2*	Leucine zipper putative tumor suppressor 2	Q6P9Q4	*FHOD1*	FH1/FH2 domain-containing protein 1
Q8K212	*PACS1*	Phosphofurin acidic cluster sorting protein 1	Q99L88	*SNTB1*	Beta-1-syntrophin
Q3U3R4	*LMF1*	Lipase maturation factor 1	Q80UZ0	*FGD5*	FYVE, RhoGEF and PH domain-containing protein 5
Q9JI46	*NUDT3*	Diphosphoinositol polyphosphate phosphohydrolase 1	O35704	*SPTLC1*	Serine palmitoyltransferase 1
Q8K4G1	*LTBP-4*	Latent-transforming growth factor beta-binding protein 4	A2ASQ1	*AGRN*	Agrin
P70429	*EVL*	Ena/VASP-like protein	Q9JIG8	*PRAF2*	PRA1 family protein 2
C0HKD8	*MFAP1a*	Microfibrillar-associated protein 1A	Q8R323	*RFC3*	Replication factor C subunit 3

This table contains the 146 proteins that displayed less than 0.83-fold down-regulation in Ang II-infused mice in three independent experiments. The protein accession number, gene name, and name of each protein are provided here. The proteins are listed in ascending order according to their fold change (the fold change can be seen in Table S2), and the top 10 down-regulated proteins appear in bold.

**Table 3:** Distribution of proteins and signaling pathways response to Ang II infusion, based on GO and KEGG analysis.

Terms	Count	*p* Value	FDR	richFactor	Accession NO.
**GO (gene ontology)**					
Gene silencing (BP)	12	0.0011	0.5010	0.1600	Q91YE5, Q9Z2D6, O88712, Q6ZQ88, Q6Y7W8, Q9CY73, Q80VL1, P15864, P43276, O55135, P42227, P68433
Monosaccharide binding (MF)	9	0.0029	0.5010	0.1698	P41317, Q8K2I4, O08528, Q60715, Q64237, Q9D136, P12382, P06745, Q9JK38
Extracellular matrix (CC)	22	0.0012	0.5010	0.1164	P07356, P11276, Q9WVH9, P97429, Q8K4G1, Q62009, P97384, A2ASQ1, C0HKD8, P06797, P41317, Q8BPB5, Q80YX1, O08746, P82198, P18406, Q61704, Q8R054, Q60754, O70503, P09242, Q9CXD9
**KEGG (kyoto encyclopedia of genes and genomes) pathways**					
Fatty acid biosynthesis	4	0.0069	0.5894	0.2857	Q5SWU9, Q8JZR0, Q8R3F5, Q91WC3
EGFR tyrosine kinase inhibitor resistance	6	0.0076	0.5894	0.1935	Q63844, Q8BMB3, P63073, Q64373, P20444, P42227
PPAR signaling pathway	8	0.0092	0.5894	0.1538	P12242, Q8CGN5, O88492, Q8JZR0, P11404, Q64516, O35488, Q91WC3
HIF-1 signaling pathway	8	0.0103	0.5894	0.1509	P12382, O08528, P11798, Q63844, Q8BMB3, P63073, P20444, P42227
Galactose metabolism	4	0.0114	0.5894	0.2500	P12382, Q9D0F9, O08528, P70699
Starch and sucrose metabolism	4	0.0143	0.6137	0.2353	Q9D0F9, O08528, P06745, P70699
Other glycan degradation	3	0.0169	0.6230	0.3000	Q8BX80, Q69ZF3, Q8K2I4
Peroxisome	8	0.0210	0.6757	0.1333	P08228, Q8JZR0, P34914, Q78JN3, Q61285, O35488, Q91WC3, Q922Z0
Lysosome	8	0.0323	0.7758	0.1231	Q61207, Q9Z1T1, P06797, O89017, P12265, P70699, O70370, Q8K2I4
Thiamine metabolism	2	0.0427	0.7758	0.3333	P09242, Q9R0Y5
Apoptosis	7	0.0443	0.7758	0.1228	P60710, O08600, P06797, Q63844, Q64373, O70370, O54788
Ras signaling pathway	8	0.0472	0.7758	0.1143	P62874, Q05144, Q9DAS9, Q63844, Q64373, P20444, P70392, Q9D4H1

Abbreviations: BP, biological processes; MF, molecular functions; CC, cellular components; FDR, false discovery rate. GO and KEGG pathway enrichment were analyzed by the Fisher’ exact test, based on the entire quantified protein annotations as the background dataset. Only functional categories and pathways with *p*-values < 0.05 were considered as significant.

**Table 4:** DEPs with high connectivity degree in PPI analysis between saline and Ang II infusion group.

Accession NO.	Gene Name	Protein Name	Degree	Up/Down Regulation
P11276	*FN1*	Fibronectin	28	up
P60710	*ACTB*	Actin, cytoplasmic 1	25	down
Q63844	*MAPK3*	Mitogen-activated protein kinase 3	17	down
Q9CRB2	*NHP2*	H/ACA ribonucleoprotein complex subunit 2	17	down
P42227	*STAT3*	Signal transducer and activator of transcription 3	16	up
P50580	*PA2G4*	Proliferation-associated protein 2G4	15	up
Q99ME9	*GTPBP4*	Nucleolar GTP-binding protein 1	14	down
O55135	*eIF6*	Eukaryotic translation initiation factor 6	14	down
P61202	*COPS2*	COP9 signalosome complex subunit 2	12	up
Q64373	*BCL2L1*	Bcl-2-like protein 1	11	down
O35488	*SLC27A2*	Very long-chain acyl-CoA synthetase	11	down
Q5SWU9	*ACACA*	Acetyl-CoA carboxylase 1	11	up
P67984	*Rpl22*	60S ribosomal protein L22	11	up
O08528	*HK2*	Hexokinase-2	10	up
P08228	*SOD1*	Superoxide dismutase [Cu-Zn]	10	up
P63073	*eIF4E*	Eukaryotic translation initiation factor 4E	10	down
P63158	*HMGB1*	High mobility group protein B1	10	up
Q99L45	*EIF2S2*	Eukaryotic translation initiation factor 2 subunit 2	10	up
Q6ZQ88	*KDM1A*	Lysine-specific histone demethylase 1A	10	up
Q9WUP7	*UCHL5*	Ubiquitin carboxyl-terminal hydrolase isozyme L5	10	up

## Data Availability

The data presented in this study are available upon request from the corresponding author.

## Supplementary Materials

The following are available online at https://www.mdpi.com/article/10.3390/biomedicines9121820/s1. Supplementary figure legends, Figure S1: statistical histogram of protein identification and quantitative results; Figure S2: quality control of peptides; Figure S3: diagram of the fatty acid biosynthesis; Figure S4: diagram of PPAR signaling pathway; Figure S5: diagram of Ras signaling pathway; Figure S6: diagram of HIF-1 signaling pathway; Figure S7: diagram of apoptosis pathway; Table S1: parameters and instructions of MASCOT engine search. Table S2: Quantitative analysis of all differentially expressed proteins.

## References

[1] Bourmoum M., Charles R., Claing A. (2016). The GTPase ARF6 controls ROS production to mediate angiotensin II-induced vascular smooth muscle cell proliferation. PLoS ONE.

[2] Nour-Eldine W., Ghantous C.M., Zibara K., Dib L., Issaa H., Itani H.A., El-Zein N., Zeidan A. (2016). Adiponectin attenuates angiotensin II-induced vascular smooth muscle cell remodeling through nitric oxide and the RhoA/ROCK pathway. Front. Pharmacol..

[3] Dai F., Qi Y., Guan W., Meng G., Liu Z., Zhang T., Yao W. (2019). RhoGDI stability is regulated by SUMOylation and ubiquitination via the AT1 receptor and participates in Ang II-induced smooth muscle proliferation and vascular remodeling. Atherosclerosis.

[4] Siegel-Axel D.I., Häring H.U. (2016). Perivascular adipose tissue: An unique fat compartment relevant for the cardiometabolic syndrome. Rev. Endocr. Metab. Disord..

[5] Fernández-Alfonso M.S., Gil-Ortega M., Aranguez I., Souza D., Dreifaldt M., Somoza B., Dashwood M.R. (2017). Role of PVAT in coronary atherosclerosis and vein graft patency: Friend or foe?. Br. J. Pharmacol..

[6] Qi X.Y., Qu S.L., Xiong W.H., Rom O., Chang L., Jiang Z.S. (2018). Perivascular adipose tissue (PVAT) in atherosclerosis: A double-edged sword. Cardiovasc. Diabetol..

[7] Langin D., Arner P. (2006). Importance of TNF alpha and neutral lipases in human adipose tissue lipolysis. Trends. Endocrinol. Metab..

[8] Cao H. (2014). Adipocytokines in obesity and metabolic disease. J. Endocrinol..

[9] Cannon B., Nedergaard J. (2004). Brown adipose tissue: Function and physiological significance. Physiol. Rev..

[10] Kern P.A., Finlin B.S., Zhu B., Rasouli N., McGehee R.E., Westgate P.M., Dupont-Versteegden E.E. (2014). The effects of temperature and seasons on subcutaneous white adipose tissue in humans: Evidence for thermogenic gene induction. J. Clin. Endocrinol. Metab..

[11] Lin J.Z., Martagón A.J., Cimini S.L., Gonzalez D.D., Tinkey D.W., Biter A., Baxter J.D., Webb P., Gustafsson J.Å., Hartig S.M. (2015). Pharmacological activation of thyroid hormone receptors elicits a functional conversion of white to brown fat. Cell. Rep..

[12] Wu J., Boström P., Sparks L.M., Ye L., Choi J.H., Giang A.H., Khandekar M., Virtanen K.A., Nuutila P., Schaart G. (2012). Beige adipocytes are a distinct type of thermogenic fat cell in mouse and human. Cell.

[13] Uysal K.T., Wiesbrock S.M., Marino M.W., Hotamisligil G.S. (1997). Protection from obesity-induced insulin resistance in mice lacking TNF-alpha function. Nature.

[14] Janke J., Engeli S., Gorzelniak K., Luft F.C., Sharma A.M. (2002). Mature adipocytes inhibit in vitro differentiation of human preadipocytes via angiotensin type 1 receptors. Diabetes.

[15] Than A., Leow M.K., Chen P. (2013). Control of adipogenesis by the autocrine interplays between angiotensin 1-7/Mas receptor and angiotensin II/AT1 receptor signaling pathways. J. Biol. Chem..

[16] Sysoeva V.Y., Ageeva L.V., Tyurin-Kuzmin P.A., Sharonov G.V., Dyikanov D.T., Kalinina N.I., Tkachuk V.A. (2017). Local angiotensin II promotes adipogenic differentiation of human adipose tissue mesenchymal stem cells through type 2 angiotensin receptor. Stem. Cell. Res..

[17] Wiśniewski J.R., Zougman A., Nagaraj N., Mann M. (2009). Universal sample preparation method for proteome analysis. Nat. Methods.

[18] Francois A.A., Obasanjo-Blackshire K., Clark J.E., Boguslavskyi A., Holt M.R., Parker P.J., Marber M.S., Heads R.J. (2018). Loss of Protein Kinase Novel 1 (PKN1) is associated with mild systolic and diastolic contractile dysfunction, increased phospholamban Thr17 phosphorylation, and exacerbated ischaemia-reperfusion injury. Cardiovasc. Res..

[19] Fang Y., Liao G., Yu B. (2019). LSD1/KDM1A inhibitors in clinical trials: Advances and prospects. J. Hematol. Oncol..

[20] Fairbanks A.J. (2019). Chemoenzymatic synthesis of glycoproteins. Curr. Opin. Chem. Biol..

[21] Farmer S.R. (2008). Molecular determinants of brown adipocyte formation and function. Genes. Dev..

[22] Moretti F., Bergman P., Dodgson S., Marcellin D., Claerr I., Goodwin J.M., DeJesus R., Kang Z., Antczak C., Begue D. (2018). TMEM41B is a novel regulator of autophagy and lipid mobilization. EMBO. Rep..

[23] Kim K.H., Won J.H., Cheng N., Lau L.F. (2018). The matricellular protein CCN1 in tissue injury repair. J. Cell. Commun. Signal..

[24] Ren B., Liu M., Ni J., Tian J. (2018). Role of selenoprotein f in protein folding and secretion: Potential involvement in human disease. Nutrients.

[25] Zhang Q.H., Yin R.X., Chen W.X., Cao X.L., Chen Y.M. (2018). Association between the TIMD4-HAVCR1 variants and serum lipid levels, coronary heart disease and ischemic stroke risk and atorvastatin lipid-lowering efficacy. Biosci. Rep..

[26] Fu X., Shah A.P., Li Z., Li M., Tamashiro K.L., Baraban J.M. (2020). Genetic inactivation of the translin/trax microRNA-degrading enzyme phenocopies the robust adiposity induced by Translin (Tsn) deletion. Mol. Metab..

[27] Hung C.S., Lin J.C. (2020). Alternatively spliced MBNL1 isoforms exhibit differential influence on enhancing brown adipogenesis. Biochim. Biophys. Acta. Gene. Regul. Mech..

[28] Zheng X., Ren B., Li X., Yan H., Xie Q., Liu H., Zhou J., Tian J., Huang K. (2020). Selenoprotein F knockout leads to glucose and lipid metabolism disorders in mice. J. Biol. Inorg. Chem..

[29] Cheng C.K., Bakar H.A., Gollasch M., Huang Y. (2018). Perivascular adipose tissue: The sixth man of the cardiovascular system. Cardiovasc. Drugs. Ther..

[30] Miao C.Y., Li Z.Y. (2012). The role of perivascular adipose tissue in vascular smooth muscle cell growth. Br. J. Pharmacol..

[31] Campbell D.J., Habener J.F. (1987). Cellular localization of angiotensinogen gene expression in brown adipose tissue and mesentery: Quantification of messenger ribonucleic acid abundance using hybridization in situ. Endocrinology..

[32] Cassis L.A., Police S.B., Yiannikouris F., Thatcher S.E. (2008). Local adipose tissue renin-angiotensin system. Curr. Hypertens. Rep..

[33] Hanzu F.A., Musri M.M., Sánchez-Herrero A., Claret M., Esteban Y., Kaliman P., Gomis R., Párrizas M. (2013). Histone demethylase KDM1A represses inflammatory gene expression in preadipocytes. Obesity.

[34] Brina D., Miluzio A., Ricciardi S., Clarke K., Davidsen P.K., Viero G., Tebaldi T., Offenhäuser N., Rozman J., Rathkolb B. (2015). eIF6 coordinates insulin sensitivity and lipid metabolism by coupling translation to transcription. Nat. Commun..

[35] Xiong Y., Wang E., Huang Y., Guo X., Yu Y., Du Q., Ding X., Sun Y. (2016). Inhibition of lysine-specific demethylase-1 (LSD1/KDM1A) promotes the adipogenic differentiation of hESCs through H3K4 methylation. Stem. Cell. Rev. Rep..

[36] Liu C., Wang J., Wei Y., Zhang W., Geng M., Yuan Y., Chen Y., Sun Y., Chen H., Zhang Y. (2020). Fat-specific knockout of Mecp2 upregulates slpi to reduce obesity by enhancing browning. Diabetes.

[37] Reilly S.M., Hung C.W., Ahmadian M., Zhao P., Keinan O., Gomez A.V., DeLuca J.H., Dadpey B., Lu D., Zaid J. (2020). Catecholamines suppress fatty acid re-esterification and increase oxidation in white adipocytes via STAT3. Nat. Metab..

[38] Xu Y., Wang N., Tan H.Y., Li S., Zhang C., Zhang Z., Feng Y. (2020). *Panax notoginseng* saponins modulate the gut microbiota to promote thermogenesis and beige adipocyte reconstruction via leptin-mediated AMPKα/STAT3 signaling in diet-induced obesity. Theranostics.

[39] Lakhani H.V., Zehra M., Pillai S.S., Puri N., Shapiro J.I., Abraham N.G., Sodhi K. (2019). Beneficial role of HO-1-SIRT1 axis in attenuating angiotensin II-induced adipocyte dysfunction. Int. J. Mol. Sci..

[40] Goel H., Melot J., Krinock M.D., Kumar A., Nadar S.K., Lip G.Y.H. (2020). Heart-type fatty acid-binding protein: An overlooked cardiac biomarker. Ann. Med..

[41] Zhuang L., Mao Y., Liu Z., Li C., Jin Q., Lu L., Tao R., Yan X., Chen K. (2021). FABP3 deficiency exacerbates metabolic derangement in cardiac hypertrophy and heart failure via PPARα pathway. Front. Cardiovasc. Med..

[42] Betz M.J., Enerbäck S. (2018). Targeting thermogenesis in brown fat and muscle to treat obesity and metabolic disease. Nat. Rev. Endocrinol..

[43] Eugene Chen Y. (2018). Editorial: The Yin and Yang of perivascular adipose tissue in vascular disease. Cardiovasc. Drugs. Ther..

[44] Liang X., Qi Y., Dai F., Gu J., Yao W. (2020). PVAT: An important guardian of the cardiovascular system. Histol. Histopathol..

[45] Dalen K.T., Schoonjans K., Ulven S.M., Weedon-Fekjaer M.S., Bentzen T.G., Koutnikova H., Auwerx J., Nebb H.I. (2004). Adipose tissue expression of the lipid droplet-associating proteins S3-12 and perilipin is controlled by peroxisome proliferator-activated receptor-gamma. Diabetes.

[46] Smith C.E., Ordovás J.M. (2012). Update on perilipin polymorphisms and obesity. Nutr. Rev..

[47] Westhoff C.C., Mrozinski J., Riedel I., Heid H.W., Moll R. (2017). Perilipin 1 is a highly specific marker for adipocytic differentiation in sarcomas with intermediate sensitivity. J. Cancer Res. Clin. Oncol..

[48] Han X., Zhu J., Zhang X., Song Q., Ding J., Lu M., Sun S., Hu G. (2018). Plin4-dependent lipid droplets hamper neuronal mitophagy in the MPTP/p-induced mouse model of Parkinson’s disease. Front. Neurosci..

[49] Bernardi R.E., Olevska A., Morella I., Fasano S., Santos E., Brambilla R., Spanagel R. (2019). The inhibition of RasGRF2, but not RasGRF1, alters cocaine reward in mice. J. Neurosci..

[50] Huang H., Fan Y., Gao Z., Wang W., Shao N., Zhang L., Yang Y., Zhu W., Chen Z., Hu J. (2019). HIF-1α contributes to Ang II-induced inflammatory cytokine production in podocytes. BMC. Pharmacol. Toxicol..

[51] Yan X., Zhao R., Feng X., Mu J., Li Y., Chen Y., Li C., Yao Q., Cai L., Jin L. (2020). Sialyltransferase7A promotes angiotensin II-induced cardiomyocyte hypertrophy via HIF-1α-TAK1 signalling pathway. Cardiovasc. Res..

[52] Sharma N.M., Haibara A.S., Katsurada K., Nandi S.S., Liu X., Zheng H., Patel K.P. (2021). Central Ang II (Angiotensin II)-mediated sympathoexcitation: Role for HIF-1α (hypoxia-inducible factor-1α) facilitated glutamatergic tone in the paraventricular nucleus of the hypothalamus. Hypertension.

[53] Qi D., Wei M., Jiao S., Song Y., Wang X., Xie G., Taranto J., Liu Y., Duan Y., Yu B. (2019). Hypoxia inducible factor 1α in vascular smooth muscle cells promotes angiotensin II-induced vascular remodeling via activation of CCL7-mediated macrophage recruitment. Cell Death Dis..

[54] Yang X., Bao M., Fang Y., Yu X., Ji J., Ding X. (2021). STAT3/HIF-1α signaling activation mediates peritoneal fibrosis induced by high glucose. J. Transl. Med..

