# Supplementary material for: Comparative Proteomic Analysis of tPVAT during Ang II Infusion
Source: Biomedicines. 2021 Dec 2;9(12):1820. doi: 10.3390/biomedicines9121820 (PMC8698607; doi:10.3390/biomedicines9121820)
Supplement: Supplementary file 1 [file biomedicines-09-01820-s001.zip › Supplementary table S1 and figure legends.pdf]

**Supplementary figure legends**

**Figure. S1 Statistical histogram of protein identification and quantitative results.** Total spectrum: the total number of secondary spectra; Matched spectrum (PSM, Peptide Spectrum Match): the total number of database matching spectra; Peptides: the total number of peptides; Unique peptides: the total number of unique peptides; Identified proteins: total number of identified proteins; Quantified proteins: identified proteins that can be quantified.

**Figure. S2 Quality control of peptides.** (A) The mass deviations of all identified peptides are mainly distributed within 10 ppm, indicating that the identification results are accurate and reliable. (B) About 65.64% of the peptides have a MASCOT score of more than 20. The median MASCOT score is 27.49. (C) The abundance ratio of most proteins is close to 1.

**Figure. S3 Diagram of the fatty acid biosynthesis.** Red boxes represent the up-regulated DEPs, and green boxes represent the down-regulated DEPs.

**Figure. S4 Diagram of PPAR signaling pathway.** Red boxes represent the up-regulated DEPs, and green boxes represent the down-regulated DEPs.

**Figure. S5 Diagram of Ras signaling pathway.** Red boxes represent the up-regulated DEPs, and green boxes represent the down-regulated DEPs.

**Figure. S6 Diagram of HIF-1 signaling pathway.** Red boxes represent the up-regulated DEPs, and green boxes represent the down-regulated DEPs. The yellow boxes represent that there are multiple proteins, and the up or down-regulated expression is inconsistent.

**Figure. S7 Diagram of apoptosis pathway.** Red boxes represent the up-regulated DEPs, and green boxes represent the down-regulated DEPs. The yellow boxes represent that there are multiple proteins, and the up or down-regulated expression is inconsistent.

**Table S1 Parameters and instructions of MASCOT engine search**

| Item | Value |
|---|---|
| Enzyme | Trypsin |
| Max Missed Cleavages | 2 |
| Fixed modifications | Carbamidomethyl (C),<br>iTRAQ 4/8plex (N-term), iTRAQ 4/8plex (K)<br>TMT 6/10/16 plex (N-term), TMT 6/10/16 plex (K) |
| Variable modifications | Oxidation (M), iTRAQ 4/8plex (Y), TMT 6/10/16 plex (Y) |
| Peptide Mass Tolerance | ± 20 ppm |
| Fragment Mass Tolerance | 0.1Da |
| Database | See the project report |
| Database pattern | Decoy |
| Peptide FDR | ≤0.01 |
| Protein Quantification | The protein ratios are calculated as the median of only unique peptides of the protein |
| Experimental Bias | Normalizes all peptide ratios by the median protein ratio. The median protein ratio should be 1 after the normalization. |
