# Supplementary figures and images for: Comparative Proteomic Analysis of tPVAT during Ang II Infusion
Source: Biomedicines. 2021 Dec 2;9(12):1820. doi: 10.3390/biomedicines9121820 (PMC8698607; doi:10.3390/biomedicines9121820)

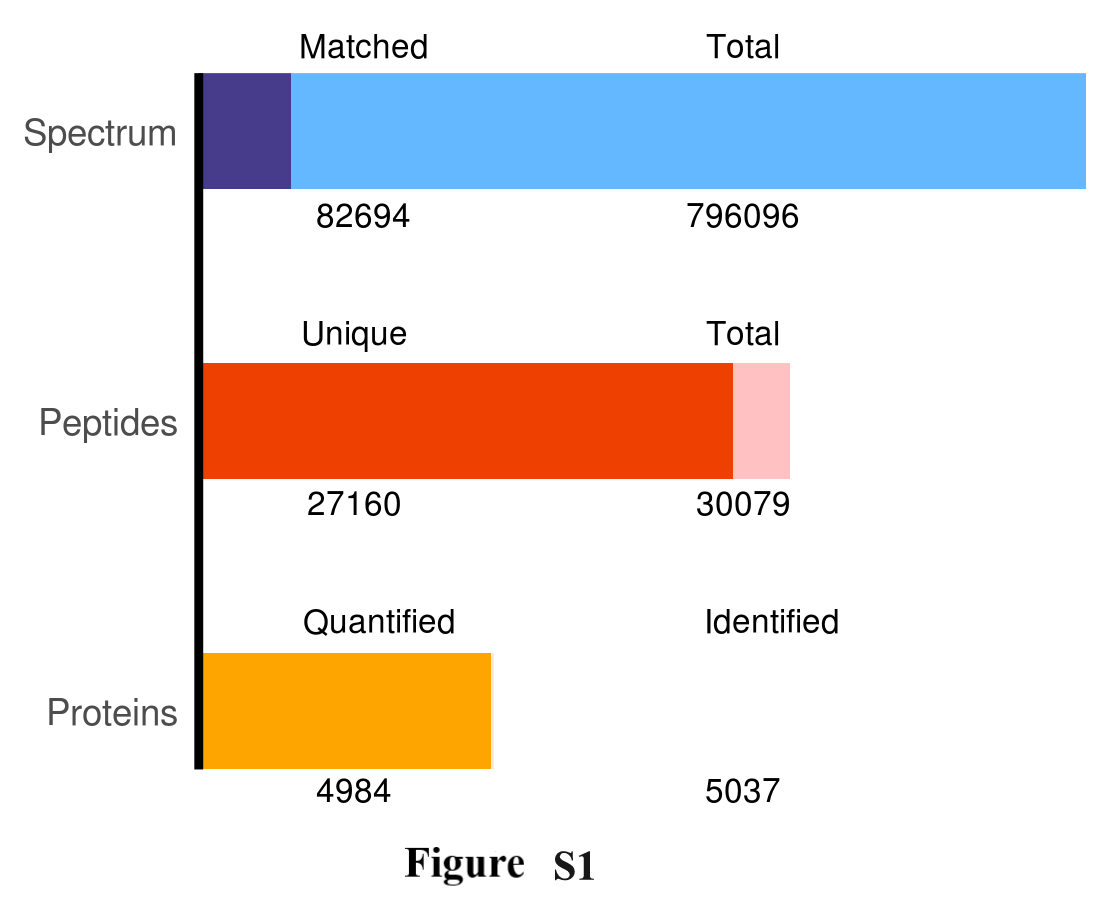

Supplement: Supplementary file 1 [file biomedicines-09-01820-s001.zip › biomedicines-1467387-suppl figures/Supplementary Figure S1.tif]

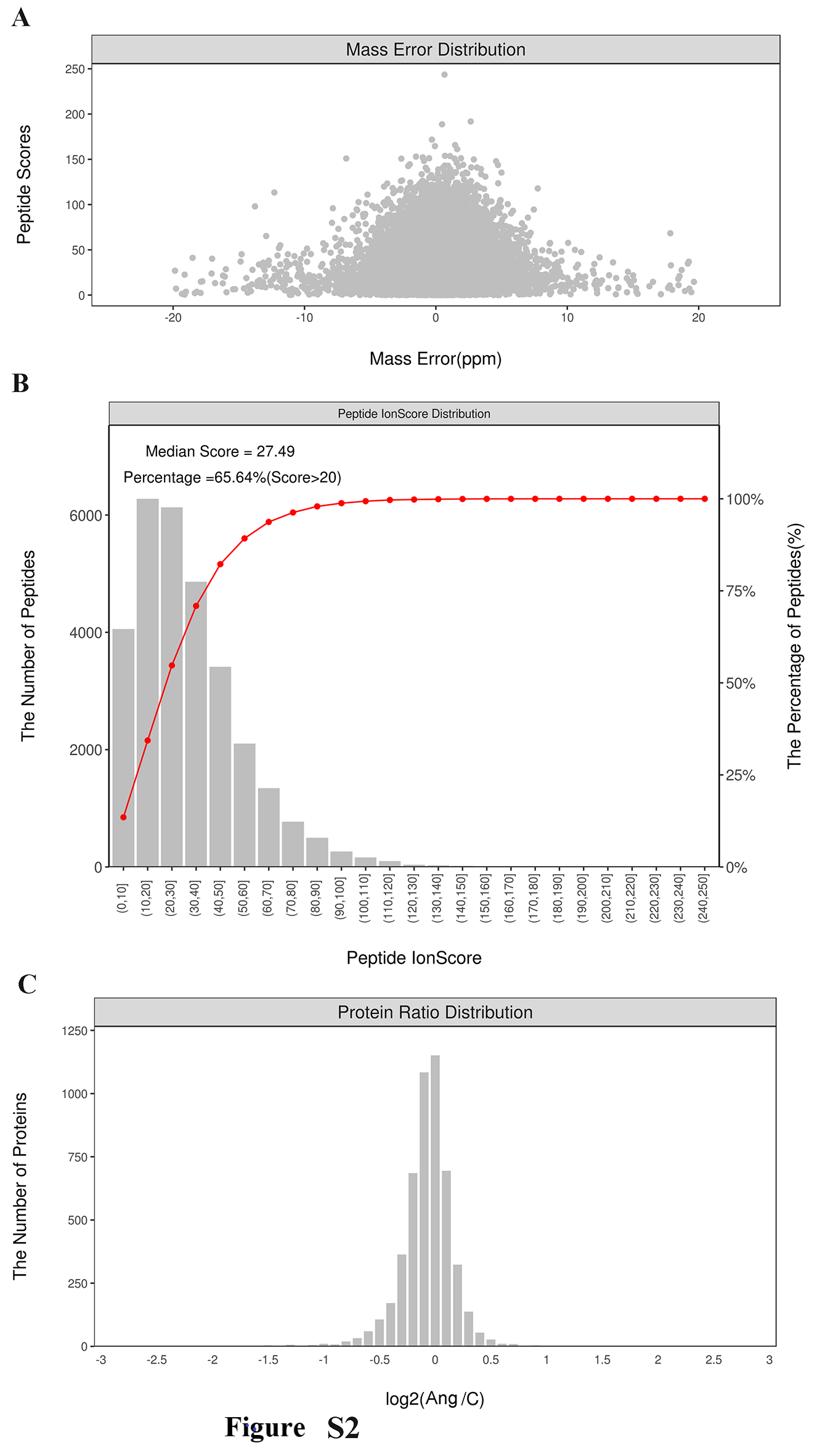

Supplement: Supplementary file 1 [file biomedicines-09-01820-s001.zip › biomedicines-1467387-suppl figures/Supplementary Figure S2.tif]

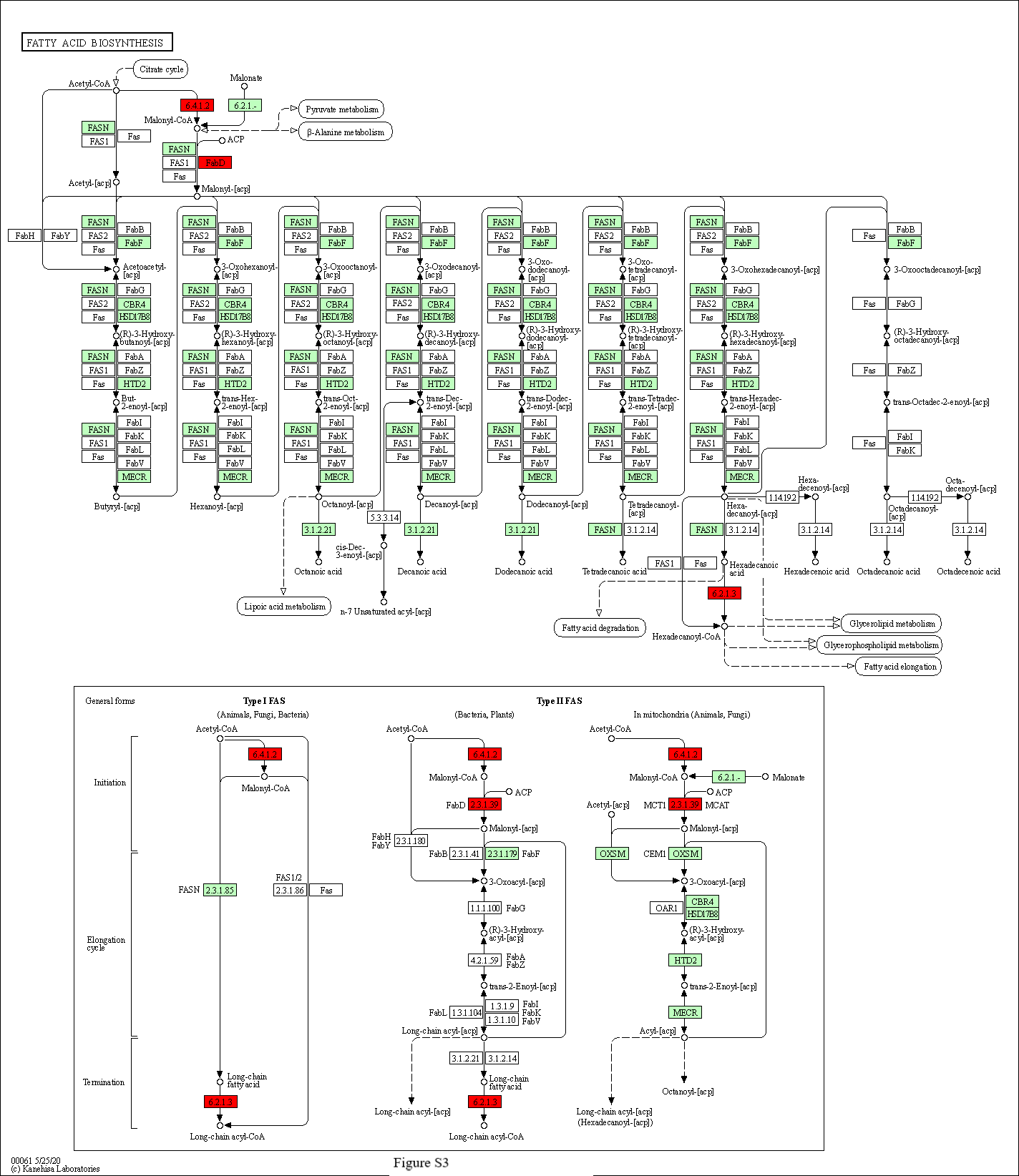

Supplement: Supplementary file 1 [file biomedicines-09-01820-s001.zip › biomedicines-1467387-suppl figures/Supplementary Figure S3.tif]

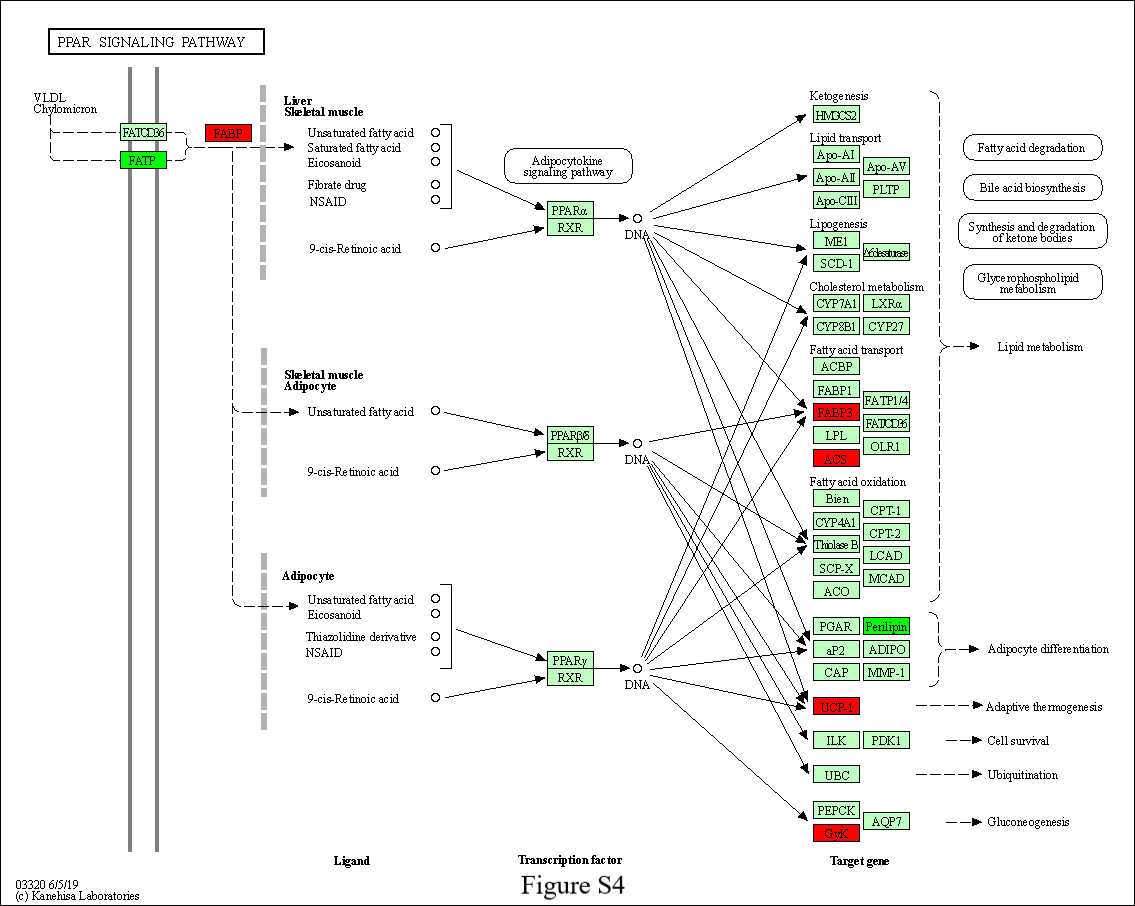

Supplement: Supplementary file 1 [file biomedicines-09-01820-s001.zip › biomedicines-1467387-suppl figures/Supplementary Figure S4.tif]

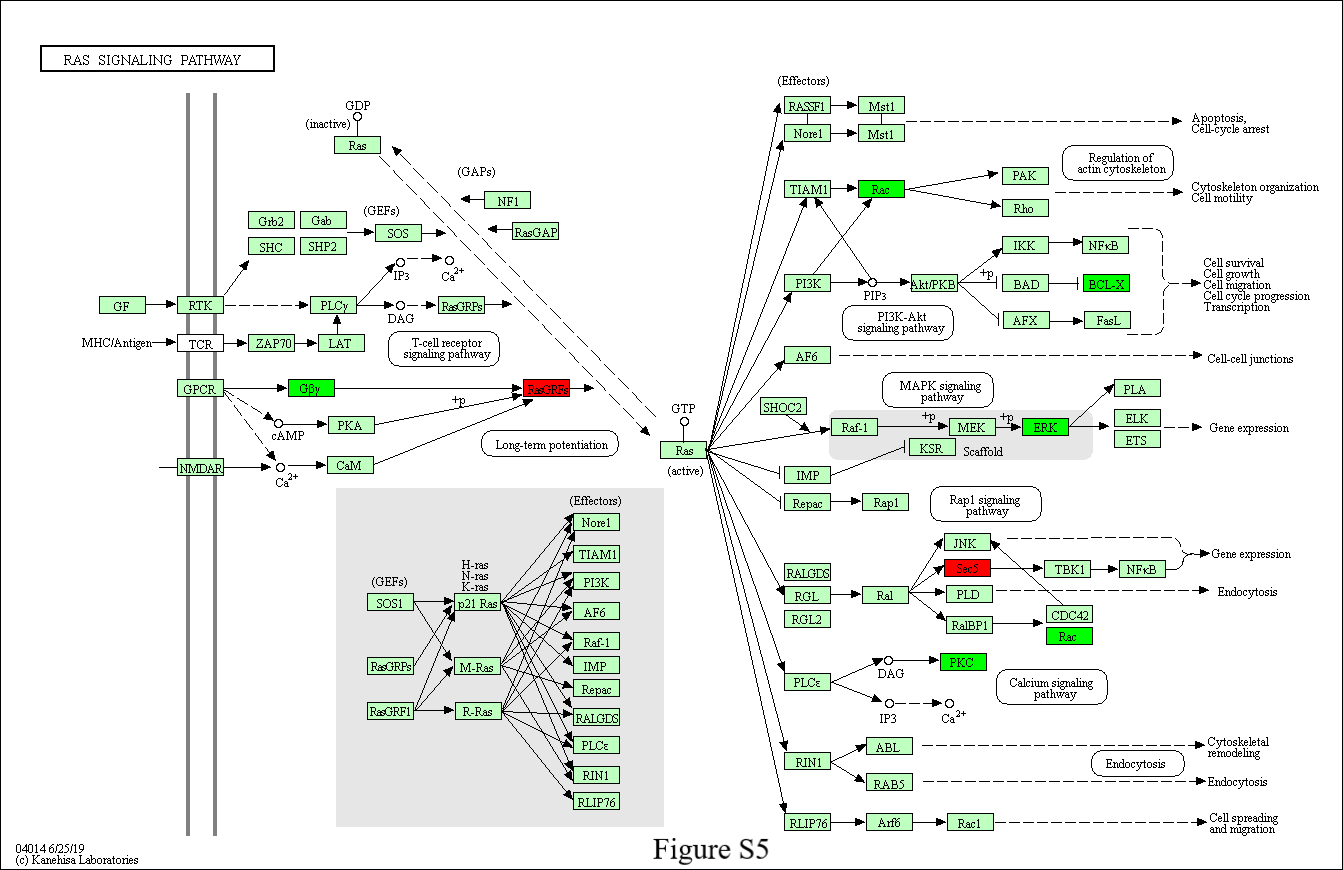

Supplement: Supplementary file 1 [file biomedicines-09-01820-s001.zip › biomedicines-1467387-suppl figures/Supplementary Figure S5.tif]

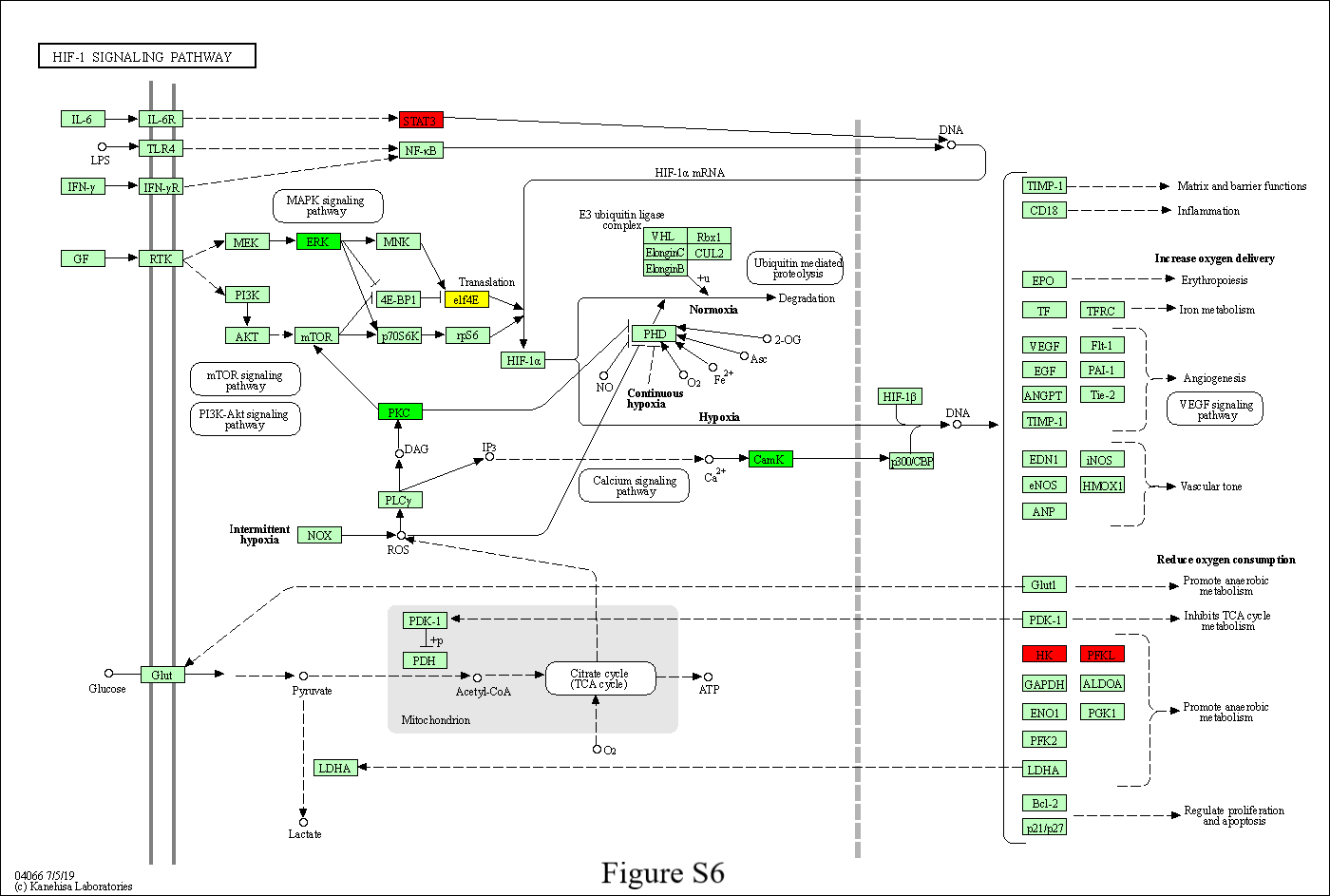

Supplement: Supplementary file 1 [file biomedicines-09-01820-s001.zip › biomedicines-1467387-suppl figures/Supplementary Figure S6.tif]

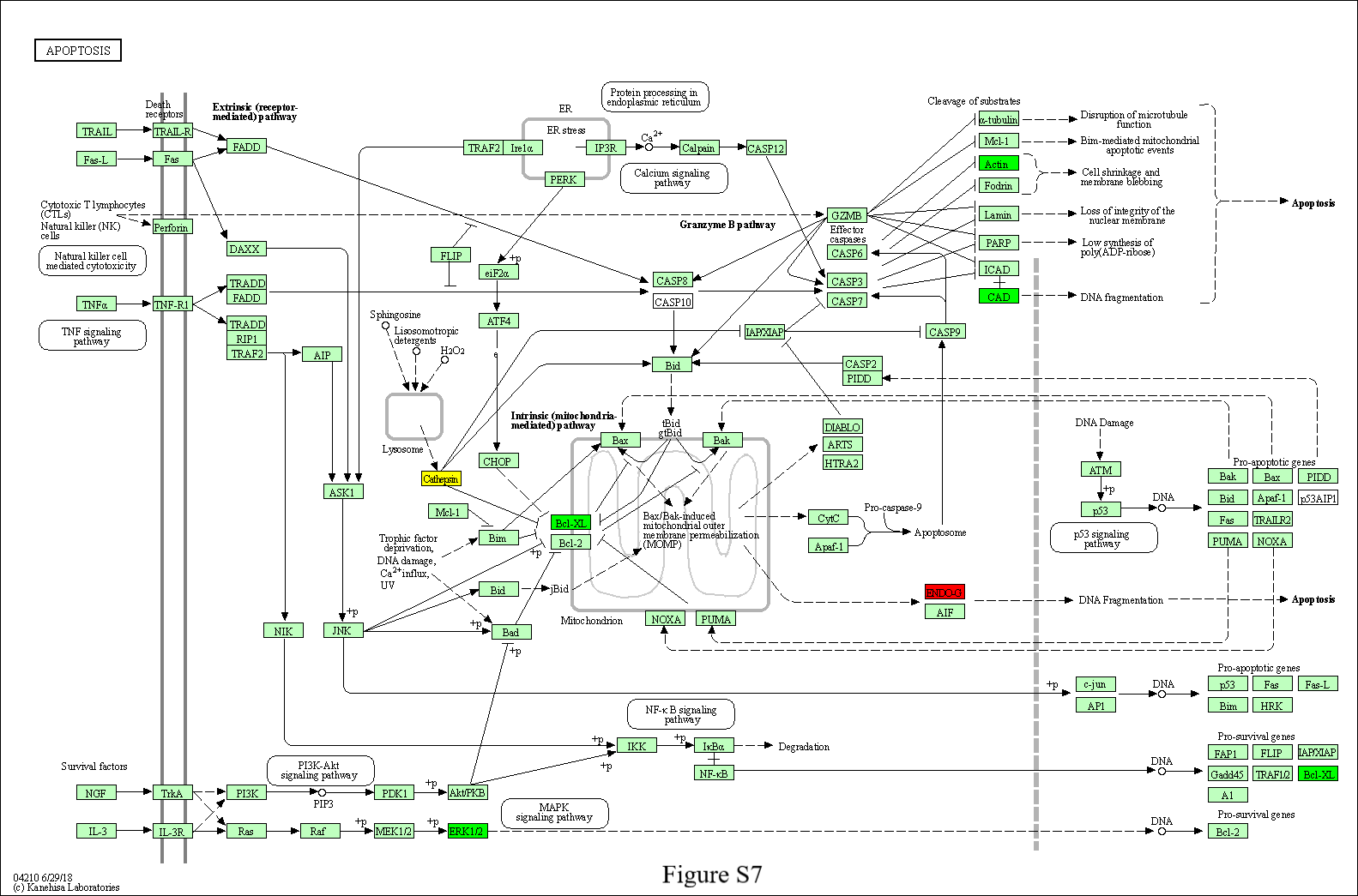

Supplement: Supplementary file 1 [file biomedicines-09-01820-s001.zip › biomedicines-1467387-suppl figures/Supplementary Figure S7.tif]
